# Comparative genomics and functional analysis of rhamnose catabolic pathways and regulons in bacteria

**DOI:** 10.3389/fmicb.2013.00407

**Published:** 2013-12-23

**Authors:** Irina A. Rodionova, Xiaoqing Li, Vera Thiel, Sergey Stolyar, Krista Stanton, James K. Fredrickson, Donald A. Bryant, Andrei L. Osterman, Aaron A. Best, Dmitry A. Rodionov

**Affiliations:** ^1^Sanford-Burnham Medical Research InstituteLa Jolla, CA, USA; ^2^Department of Biochemistry and Molecular Biology, Pennsylvania State University, University ParkPA, USA; ^3^Pacific Northwest National Laboratory, Biological Sciences DivisionRichland, WA, USA; ^4^Department of Biology, Hope CollegeHolland, MI, USA; ^5^Department of Chemistry and Biochemistry, Montana State UniversityBozeman, MT, USA; ^6^A.A. Kharkevich Institute for Information Transmission Problems, Russian Academy of SciencesMoscow, Russia

**Keywords:** L-rhamnose catabolism, metabolic reconstruction, regulon, comparative genomics, *Chloroflexus*

## Abstract

L-rhamnose (L-Rha) is a deoxy-hexose sugar commonly found in nature. L-Rha catabolic pathways were previously characterized in various bacteria including *Escherichia coli*. Nevertheless, homology searches failed to recognize all the genes for the complete L-Rha utilization pathways in diverse microbial species involved in biomass decomposition. Moreover, the regulatory mechanisms of L-Rha catabolism have remained unclear in most species. A comparative genomics approach was used to reconstruct the L-Rha catabolic pathways and transcriptional regulons in the phyla Actinobacteria, Bacteroidetes, Chloroflexi, Firmicutes, Proteobacteria, and Thermotogae. The reconstructed pathways include multiple novel enzymes and transporters involved in the utilization of L-Rha and L-Rha-containing polymers. Large-scale regulon inference using bioinformatics revealed remarkable variations in transcriptional regulators for L-Rha utilization genes among bacteria. A novel bifunctional enzyme, L-rhamnulose-phosphate aldolase (RhaE) fused to L-lactaldehyde dehydrogenase (RhaW), which is not homologous to previously characterized L-Rha catabolic enzymes, was identified in diverse bacteria including Chloroflexi, Bacilli, and Alphaproteobacteria. By using *in vitro* biochemical assays we validated both enzymatic activities of the purified recombinant RhaEW proteins from *Chloroflexus aurantiacus* and *Bacillus subtilis*. Another novel enzyme of the L-Rha catabolism, L-lactaldehyde reductase (RhaZ), was identified in Gammaproteobacteria and experimentally validated by *in vitro* enzymatic assays using the recombinant protein from *Salmonella typhimurium*. *C. aurantiacus* induced transcription of the predicted L-Rha utilization genes when L-Rha was present in the growth medium and consumed L-Rha from the medium. This study provided comprehensive insights to L-Rha catabolism and its regulation in diverse Bacteria.

## Introduction

L-rhamnose (L-Rha) is a deoxy-hexose sugar commonly found in plants as a part of complex pectin polysaccharides and in many bacteria as a common component of the cell wall (Buttke and Ingram, 1975; Giraud and Naismith, 2000). Many microorganisms including the *Enterobacteriaceae* and *Rhizobiaceae* are capable of utilizing L-Rha as a carbon source (Eagon, 1961). Plant-pathogenic species (such as *Erwinia* spp.) and saprophytic species (e.g., *Bacillus subtilis*) are able to degrade rhamnogalacturonans and other L-Rha-containing polysaccharides by a set of extracellular enzymes including rhamnogalacturonate lyases (termed RhiE in *Erwinia* spp.) and α-L-rhamnosidases (RhmA, RamA) (Laatu and Condemine, 2003; Ochiai et al., 2007; Avila et al., 2009). The resulting L-Rha and unsaturated rhamnogalacturonides can enter the cells by specific transport systems, the L-rhamnose permease RhaT in Enterobacteriaceae (Muiry et al., 1993), and the RhiT transporter in *Erwinia chrysanthemi* (Hugouvieux-Cotte-Pattat, 2004). In the latter species, the unsaturated galacturonyl hydrolase RhiN is used to release L-Rha and unsaturated galacturonate residues to promote their further catabolism in the cytoplasm (Hugouvieux-Cotte-Pattat, 2004; Rodionov et al., 2004).

**Figure 1:**
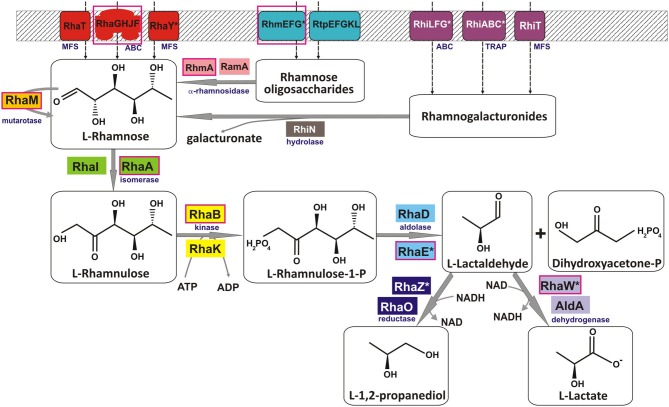
**Reconstruction of the L-rhamnose utilization pathways in bacteria.** Solid gray arrows indicate enzymatic reactions, and broken arrows denote transport. Enzyme classes and families of transporters are shown in blue subscript. Multiple non-orthologous variants of proteins for several functional roles are highlighted by the same background color. Tentatively predicted functional roles are marked by asterisks. Components of a pathway variant present in *C. aurantiacus* are shown in red boxes.

The canonical phosphorylated catabolic pathway for L-Rha described in enterobacteria is comprised of three enzymes, L-Rha isomerase (RhaA), L-rhamnulose kinase (RhaB), and L-rhamnulose-1-phosphate aldolase (RhaD) (Schwartz et al., 1974), which convert L-Rha to dihydroxyacetone phosphate (DHAP) and L-lactaldehyde (Akhy et al., 1984; Badia et al., 1989) (Figure 1). In addition, L-Rha mutarotase (RhaM) facilitates the interconversion of α and β anomers of L-Rha, providing the stereochemically less-favored anomer for the subsequent catabolic reactions (Richardson et al., 2008). The structures and reaction mechanisms each of these four enzymes from *Escherichia coli* have been determined (Korndorfer et al., 2000; Kroemer et al., 2003; Ryu et al., 2005; Grueninger and Schulz, 2006). Another L-Rha isomerase with broad substrate specificity (RhaI, 17% sequence identity to RhaA from *E. coli*) has been characterized in *Pseudomonas stutzeri* (Leang et al., 2004; Yoshida et al., 2007). L-lactaldehyde is a common product of both the L-rhamnose and L-fucose catabolic pathways and is further metabolized to L-lactate by the aldehyde dehydrogenase AldA or to 1,2-propanediol by the lactaldehyde reductase RhaO/FucO under certain conditions (Baldoma and Aguilar, 1988; Zhu and Lin, 1989; Patel et al., 2008). An alternative nonphosphorylated catabolic pathway for L-Rha comprising four metabolic enzymes L-rhamnose-1-dehydrogenase, L-rhamnono-γ-lactonase, L-rhamnonate dehydratase and L-2-keto-3-deoxyrhamnonate aldolase, by which L-Rha is converted to pyruvate and L-lactaldehyde, have been identified in fungi and two bacterial species, *Azotobacter vinelandii* and *Sphingomonas* sp. (Watanabe et al., 2008; Watanabe and Makino, 2009).

Induction of the L-Rha utilization genes in *E. coli* is mediated by two rhamnose-responsive positive transcription factors (TFs) from the AraC family, RhaS, and RhaR (Tobin and Schleif, 1990; Egan and Schleif, 1993; Via et al., 1996). RhaR activates the *rhaSR* genes via binding to the inverted repeat of two 17 bp half sites separated by a 17 bp spacer. RhaS activates the *rhaBAD* and *rhaT* genes via binding to another inverted repeat of two sites whose sequence differs from the RhaR consensus binding site. In another bacterium, the plant pathogen *Erwinia chrysanthemi* from the order *Enterobacteriales*, the expanded RhaS regulon includes a similar set of genes involved in L-Rha utilization, as well as the rhamnogalacturonides utilization genes *rhiTN* (Hugouvieux-Cotte-Pattat, 2004). The L-Rha catabolic gene cluster in *Bacteroides thetaiotaomicron* is positively controlled by another AraC-family TF, which is non-orthologous to *E. coli* RhaR (16% identity) (Patel et al., 2008). In *Rhizobium leguminosarum* bv. trifolii, a novel negative TF of the DeoR family has been implicated in control of the L-Rha utilization regulon, which contains two divergently transcribed operons, *rhaRSTPQUK* and *rhaDI*, encoding an ABC transporter for L-Rha uptake (RhaSTPQ), an alternative kinase (RhaK, 19% identity to RhaB from *E. coli*), an isomerase (RhaI), and a mutarotase (RhaU, 41% identity to RhaM from *E. coli*) (Richardson et al., 2004, 2008; Richardson and Oresnik, 2007).

Our initial genome analysis suggested the presence of a novel variant of the L-Rha utilization pathway in anoxygenic phototrophic bacteria from the *Chloroflexi* phylum. Indeed, the existence of such pathway was implicated by the presence of *rhaA* and *rhaB* gene orthologs and the absence of *rhaD* and *rhaO* genes in *Chloroflexus aurantiacus*. Moreover, the L-Rha catabolic pathway is not completely understood in many more bacterial species including *Bacillus subtilis*, and *Streptomyces coelicolor*. Mechanisms of transcriptional regulation of L-Rha utilization genes are also poorly understood in many species beyond the models. With the availability of hundreds of sequenced bacterial genomes, it is possible to use comparative genomics to reconstruct metabolic pathways and regulatory networks in individual taxonomic groups of Bacteria (Rodionov et al., 2010, 2011; Ravcheev et al., 2011, 2013; Leyn et al., 2013). Genome context-based techniques, including the analysis of chromosomal gene clustering, protein fusion events, phylogenetic co-occurrence profiles, and the genomic inference of metabolic regulons, are highly efficient methods for elucidation of novel sugar catabolic pathways. In our previous studies, we combined the genomic reconstruction of metabolic and regulatory networks with experimental testing of selected bioinformatic predictions to map sugar catabolic pathways systematically in two diverse taxonomic groups of bacteria, *Shewanella*, and *Thermotoga* (Rodionov et al., 2010, 2013). Furthermore, we have applied the integrated bioinformatic and experimental approaches to predict and validate novel metabolic pathways and transcriptional regulons involved in utilization of arabinose (Zhang et al., 2012), xylose (Gu et al., 2010), N-acetylglucosamine (Yang et al., 2006), N-acetylgalactosamine (Leyn et al., 2012), galacturonate (Rodionova et al., 2012a), and inositol (Rodionova et al., 2013) in diverse bacterial lineages.

In this work, we combined genomics-based reconstruction of L-Rha utilization pathways and RhaR transcriptional regulons in bacteria from diverse taxonomic lineages with the experimental validation of the L-Rha utilization system in *C. aurantiacus* and two other microorganisms. A novel bifunctional enzyme (named RhaEW) catalyzing two consecutive steps in L-Rha catabolism, L-rhamnulose-phosphate aldolase and L-lactaldehyde dehydrogenase, was identified in diverse bacterial lineages including Actinobacteria, α-proteobacteria, Bacilli, Bacteroidetes, and Chloroflexi. The predicted dual function of RhaEW was validated by *in vitro* enzymatic assays with recombinant proteins from *C. aurantiacus* and *B. subtilis*. Another enzyme involved in L-lactaldehyde utilization in γ-proteobacteria, L-lactaldehyde reductase RhaZ, was identified and experimentally confirmed in *Salmonella* spp. Comparative analyses of upstream regions of the L-Rha utilization genes allowed identification of candidate DNA motifs for various groups of regulators from different TF families and reconstruction of putative rhamnose regulons. L-Rha-specific transcriptional induction and the predicted DNA binding motif of a novel DeoR-family regulator for of the *rha* genes were experimentally confirmed in *C. aurantiacus*.

## Materials and methods

### Genomic reconstruction of rhamnose utilization pathways and regulons

The comparative genomic analysis of L-Rha utilization subsystem was performed using the SEED genomic platform (Overbeek et al., 2005), which allowed annotation and capture of gene functional roles, their assignment to metabolic subsystems, identification of non-orthologous gene displacements, and projection of the functional annotations across microbial genomes, as it was previously described for other sugar catabolic subsystems (Rodionov et al., 2010, 2013; Leyn et al., 2012; Rodionova et al., 2012a, 2013). The obtained functional gene annotations were captured in the SEED subsystem available online at http://pubseed.theseed.org/SubsysEditor.cgi?page=ShowSubsystem&subsystem=L-rhamnose_utilization and are summarized in Table S1 in the Supplementary Material.

For reconstruction of RhaR regulons we used an established comparative genomics approach based on identification of candidate regulator-binding sites in closely related bacterial genomes implemented in the RegPredict Web server tool (regpredict.lbl.gov) (Novichkov et al., 2010). First, we identified potential *rhaR* transcription factor genes that are located within the conserved neighborhoods of the L-Rha catabolic genes in bacterial genomes from each studied taxonomic lineage. Identification of orthologs in closely related genomes and gene neighborhood analysis were performed in MicrobesOnline (http://microbesonline.org/) (Dehal et al., 2010). To find the conserved DNA-binding motifs for each group of orthologous RhaR regulators, we used initial training sets of genes that are co-localized with *rhaR* orthologs (putative operons containing at least one candidate L-Rha utilization gene and that are located in the vicinity of a maximum ten genes from a *rhaR* gene), and then we updated each set by the most likely RhaR-regulated genes confirmed by the comparative genomics tests as well as functional considerations (i.e., involvement of candidate target genes in the L-Rha utilization pathway). Using the Discover Profile procedure in RegPredict, common DNA motifs with palindromic or direct repeat symmetry were identified and their corresponding position weight matrices (PWMs) were constructed. The initial PWMs were used to scan the reference genomes and identify additional RhaR-regulated genes that share similar binding sites in their upstream regions. The conserved regulatory interactions were included in the reconstructed RhaR regulons using the clusters of co-regulated orthologous operons in RegPredict. Candidate sites associated with new members of the regulon were added to the training set, and the respective lineage-specific PWM was rebuilt to improve search accuracy. Sequence logos for the derived DNA-binding motifs were built using the Weblogo package (Crooks et al., 2004). The details of all reconstructed regulons are displayed in the RegPrecise database of regulons (Novichkov et al., 2013) available online at http://regprecise.lbl.gov/RegPrecise/collection_pathway.jsp?pathway_id=34.

### Gene cloning and protein purification

The *rhaEW* (*Caur_2283*) and *rhaR* (*Caur_2290*) genes from *C. aurantiacus* J-10-fl, the *rhaEW* (*yuxG*) gene from *B. subtilis*, and the *rhaZ* (*STM4044*) and *rhaD* (*STM4045*) genes from *Salmonella enterica* serovar Typhimurium LT2 were amplified by PCR from genomic DNA using specific primer pairs (see Table S2 in Supplementary Material). A pET-derived vector, pODC29 Gerdes et al. (2006), containing a T7 promoter and an N-terminal His_6_ tag, or a similar vector, pProEX HTb (Invitrogen), with a *trc* promoter was used for cloning and protein expression. The *rhaR* gene was cloned into the pSMT3 expression vector (Mossessova and Lima, 2000) (a kind gift of Dr. Lima from Cornell University). The obtained plasmid encodes a fusion between the RhaR protein and an N-terminal Hexa-histidine Smt3 polypeptide (a yeast SUMO ortholog), which enhances protein solubility. The resulting plasmids were transformed into *E. coli* BL21/DE3 or BL21 (Gibco-BRL, Rockville, MD). Recombinant proteins were overexpressed as fusions with an N-terminal His_6_tag and purified to homogeneity using Ni^2+^-chelation chromatography. Cells were grown in LB medium (50 ml), induced by addition of 0.2 mM isopropyl-β-D-thiogalactopyranoside, and harvested after 4 h of additional shaking at 37°C (for Caur_2283, and Caur_2290) or 16 h of shaking at 25°C (for YuxG, STM4044, and STM4045). Harvested cells were resuspended in 20 mM HEPES buffer (pH 7) containing 100 mM NaCl, 0.03% Brij-35, 2 mM β-mercaptoethanol, and 2 mM phenylmethylsulfonyl fluoride (Sigma-Aldrich). Cells were lysed by incubation with lysozyme (1 mg/ml) for 30 min, followed by a freeze-thaw cycle and sonication. After centrifugation, Tris-HCl buffer (pH 8) was added to the supernatant (50 mM, final concentration), which was loaded onto Ni-nitrilotriacetic acid (NTA) agarose minicolumn (0.3 ml) from Qiagen Inc. (Valencia, CA). After washing with starting buffer containing 1 M NaCl and 0.3% Brij-35 bound proteins were eluted with 0.3 ml of the same buffer supplemented with 250 mM imidazole. The purified proteins were electrophoresed on a 12% (w/v) sodium dodecyl sulfate-polyacrylamide gel to monitor size and purity (>90%). Protein concentration was determined by the Quick Start Bradford Protein Assay kit from Bio-Rad.

### Enzyme assays

Aldolase/dehydrogenase activities of the purified recombinant RhaEW proteins from *C. aurantiacus* (*Ca*_RhaEW) and *B. subtilis* (*Bs*_RhaEW), and the *St*_RhaD and *St*_RhaZ proteins from *Salmonella typhimurium* were tested by a direct NADH detection assay. Because L-rhamnulose-1-P is not commercially available, we used an enzymatic coupling assay with two upstream catabolic enzymes for the conversion of L-Rha to L-rhamnulose-1-P. The L-Rha isomerase RhaA from *E. coli* (*Ec*_RhaA) and the L-rhamnulose kinase RhaB from *Thermotoga maritima* (*Tm*_RhaB) were expressed in *E. coli* and purified as described previously (Rodionova et al., 2012b). For *Ca*_RhaEW assays, the purified recombinant enzymes, *Ec*_RhaA (2 μ g) and *Tm*_RhaB (2 μ g), were pre-incubated during 20 min at 37°C in 100 μl of reaction mixture containing 150 mM Tris-HCl (pH 8), 20 mM MgCl_2_, 10 mM ATP, 1.4 mM NAD^+^, 10 μ M ZnSO_4_, and 8 mM L-Rha. Then *Ca*_RhaEW (0.5 μ g) was added to the assay mixture and the reduction of NAD^+^ was followed by increase in absorbance at 340 nm at different temperatures (30–70°C) in the spectrophotometer. For *Bs*_RhaEW, *St*_RhaD and *St*_RhaZ assays, *Ec*_RhaA and *Tm*_RhaB were pre-incubated in a ratio of 40:1 (RhaA:RhaB) at 25°C for 40 min in a reaction mixture containing 50 mM Tris-HCl (pH 7.5), 20 mM MgCl_2_, 1 mM ATP, 50 mM KCl, 2.5 mM NAD^+^ or 0.25 mM NADH, 5 μ M ZnCl_2_, and 2 mM L-Rha. Subsequently, either *Bs*_RhaEW (2.8 μ g) or *St*_RhaD (10 μ g) and *St*_RhaZ (2.8 μ g) enzymes were added and the reduction of NAD^+^ or oxidation of NADH was monitored by increase or decrease in absorbance at 340 nm, respectively, at 25°C in a final reaction volume of 200 μl.

### GC-MS analysis

Four-step biochemical conversions of L-Rha to L-lactate and DHAP by mixtures of the three L-Rha catabolic enzymes were monitored by GC-MS. Samples from enzymatic assay mixtures (10 μl) were dried in a vacuum centrifuge at room temperature, and derivatized at 80°C for 20 min with 75 μl of pyridine containing 50 mg ml^−1^ methoxylamine or ethylhydroxylamine (for lactate detection). The solution was incubated at 80°C for 60 min with 75 μl of N,O-*bis*-(trimethylsilyl)trifluoroacetamide or N-*tert*-butyldimethylsilyl-N-methyltrifluoroacetamide (for lactate detection). After derivatization, the samples were centrifuged for 1 min at 14,000 r.p.m. and the supernatant (1 μl) was transferred to vials for GC-MS analysis. A QP2010 Plus GC-MS instrument was from Shimadzu (Columbia, MD). GC-MS analyses were performed as previously described in Rodionova et al. (2012a, 2013).

### Bacterial strains and growth conditions

The *yuxG*(*rhaEW*) and *yceI*(*niaP*) disruption strains of *B. subtilis* were obtained from the joint Japanese and European *B. subtilis* consortium (Kobayashi et al., 2003). The latter strain with an insertion in the niacin transporter *niaP* was used as an isogenic negative control. Both strains were grown overnight at 37°C in chemically defined medium containing D-glucose (4 g/l), L-tryptophan (50 mg/l), L-glutamine (2 g/l), K_2_HPO_4_ (10 g/l), KH_2_PO_4_ (6 g/l), sodium citrate (1 g/l), MgSO_4_ (0.2 g/l), K_2_SO_4_ (2 g/l), FeCl_3_ (4 mg/l), and MnSO_4_ (0.2 mg/l) in the presence of erythromycin (0.5 mg/l) (pMUTIN2 marker). Overnight cultures were diluted ~10-fold to yield the same cell density (optical density at 600 nm of 0.05) in the defined medium lacking glucose and washed three times to remove residual glucose. Cells were grown in triplicate in one of two versions of the defined medium containing L-Rha (4 g/l), or no additional carbon source. *C. aurantiacus* J-10-fl was grown at 52°C in 25 ml screw capped glas tubes completely filled with BG-11 medium (Stanier et al., 1971) supplemented with 0.02% (w/v) of NH_4_Cl and 2 mM of NaHCO_3_. 0.2% of yeast extract (YE) or 35 mM of pyruvate, both with and without additional 20 mM L-Rha, were used as main carbon source and cultures grown under microaerobic starting conditions in the light. Cultures were constantly mixed on a rotation wheel during incubation. Growth of cultures was monitored at 600 nm using a ELX-808IU microplate reader from BioTek Instruments Inc. (Winooski, VT). The concentration of L-Rha in culture fluids was determined on an HPLC equipped with an HPX 78 (Bio-Rad) column.

### RT-PCR

Individual transcript levels were measured for seven genes from *C. aurantiacus*: *rhaB* (*Caur_2282*), *rhaF* (*Caur_2286*), *rhaR* (*Caur_2290*), *rhmA* (*Caur_0361*), and *Caur_0839* (NADH-flavin oxidoreductase/NADH oxidase). The latter housekeeping gene was used as a positive control since it was found to be highly expressed under both photoheterotrophic as well as chemoheterotrophic conditions in a previous proteome study (Cao et al., 2012). Total RNA was isolated from cells grown on BG-11 medium supplied with YE, YE plus L-Rha, pyruvate, and pyruvate plus L-Rha under suboxic conditions in the light, and collected after 3 days at optical densities at 650 nm of 1.3, 0.9, 0.4, and 0.6, respectively. RNA was isolated using a phenol-chloroform extraction method adapted from (Aiba et al., 1981; Steunou et al., 2006). Cell pellets were resuspended in 250 μl 10 mM sodium acetate (pH 4.5) and 37.5 μl 500 mM Na_2_EDTA (pH 8.0), then mixed with 375 μl Lysis buffer (10 mM sodium acetate, 2% SDS, pH to 4.5). Hot (65°C) acidic (pH 4.5) phenol (700 μl) was added, the sample was vortexed and incubated at 65°C for 3 min. After centrifugation (17,000 × g, 2 min), the RNA was further purified by one phenol-chloroform-isoamyl alcohol (25:24:1) and one chloroform extraction. RNA was precipitated using 0.1 volume of 10 M LiCl and 2.5 volume 100% EtOH and precipitated at -80°C for at least 30 min, washed with 80% EtOH and resuspended in DEPC treated H_2_O. The RNA solution was treated with DNase I (New England Biolab Inc.) and re-precipitated after an additional chloroform:isoamyl alcohol (24:1) extraction. The purified RNA was dissolved in DEPC-treated water. Semi-quantitative RT-PCR was conducted using a Bioline Tetro one-step RT-PCR kit following the manufacturer's protocol. The gene-specific primers for each gene tested are shown in Table S2 in Supplementary Material. For each reaction one control for DNA contamination was included (same template as for RT-PCR, started with inactivation of RT-Polymerase step) and a PCR positive control (using 10 ng whole genome DNA from *C. aurantiacus* as template) was used. PCR conditions were the same for each primer pair used. All started with a 30 min RT-step at 42°C followed by an RT-inactivation step at 95°C. Then a single step PCR for amplification of the genes from cDNA was conducted using 30 cycles of 30 s denaturation at 95°C, 30 s annealing at 60°C, and 90 s elongation step at 72°C before cooling down to 10°C.

### DNA binding assays

The interaction of the purified recombinant *C. aurantiacus* RhaR protein with its cognate DNA binding site in *C. aurantiacus* was assessed using an electrophoretic mobility-shift assay (EMSA). The His_6_-Smt3-tag was cleaved from the purified RhaR protein by digestion with Ulp1 protease. Complementary DNA fragments, containing the predicted 38-bp RhaR binding site from the *Caur_2290* promoter region and flanked on each side by five guanosine residues (Table S2 in Supplementary Materials) were synthesized by Integrated DNA Technologies. One strand of oligo was 3'-labeled by a biotin label, whereas the complementary oligo was unlabeled. Double-stranded labeled DNA fragments were obtained by annealing the labeled oligonucleotides with unlabeled complementary oligonucleotides at a 1:10 ratio. The biotin-labeled 48-bp DNA fragment (0.2 nM) was incubated with increasing concentrations of the purified RhaR protein (10–1000 nM) in a total volume of 20 μl of the binding buffer containing 50 mM Tris-HCl (pH 8.0), 150 mM NaCl, 5 mM MgCl_2_, 1 mM DDT, 0.05% NP-40, and 2.5% glycerol. Poly(dI-dC) (1 μ g) was added as a nonspecific competitor DNA to reduce non-specific binding. After 25 min of incubation at 50°C, the reaction mixtures were separated by electrophoresis on a 1.5% (w/v) agarose gel at room temperature. The DNA was transferred by electrophoresis onto a Hybond-N^+^ membrane and fixed by UV-cross-linking. The biotin-labeled DNA was detected with the LightShift chemiluminiscent EMSA kit (Thermo Fisher Scientific Inc, Rockford, IL, USA). Additional DNA fragment of the *Caur_0003* gene upstream region (Table S2 in Supplementary Materials) was used as a negative control. The effect of D-glucose, L-Rha, and L-rhamnulose (obtained by enzymatic conversion of L-Rha by *Ec*_RhaA) was tested by their addition to the incubation mixture.

## Results

### Comparative genomics of L-rhamnose utilization in bacteria

To reconstruct catabolic pathways and transcriptional regulons involved in L-Rha utilization in bacteria we utilized the subsystem-based comparative genomics approach implemented in the RegPredict and the SEED Web resources (Overbeek et al., 2005; Novichkov et al., 2010). As a result, the L-Rha metabolic pathway genes and transcriptional regulons were identified in complete genomes of 55 representatives of diverse taxonomic groups of bacteria including the *Actinomycetales*, *Bacteroiodales*, *Chloroflexales*, *Bacillales*, *Rhizobiales*, *Enterobacteriales*, and *Thermotogales*. The distribution of genes encoding the L-Rha catabolic enzymes and associated transporters and regulators across the studied species is summarized in Table S1 in Supplementary Material. The studied bacterial species possess many variations in key enzymes from the L-Rha catabolic pathway, as well as in mechanisms of sugar uptake and transcriptional regulation. Some of these variations are briefly described below when we describe novel functional variants of the L-Rha catabolic pathway and novel transcriptional regulons for these pathways.

#### L-rhamnose catabolic regulons

**Figure 2:**
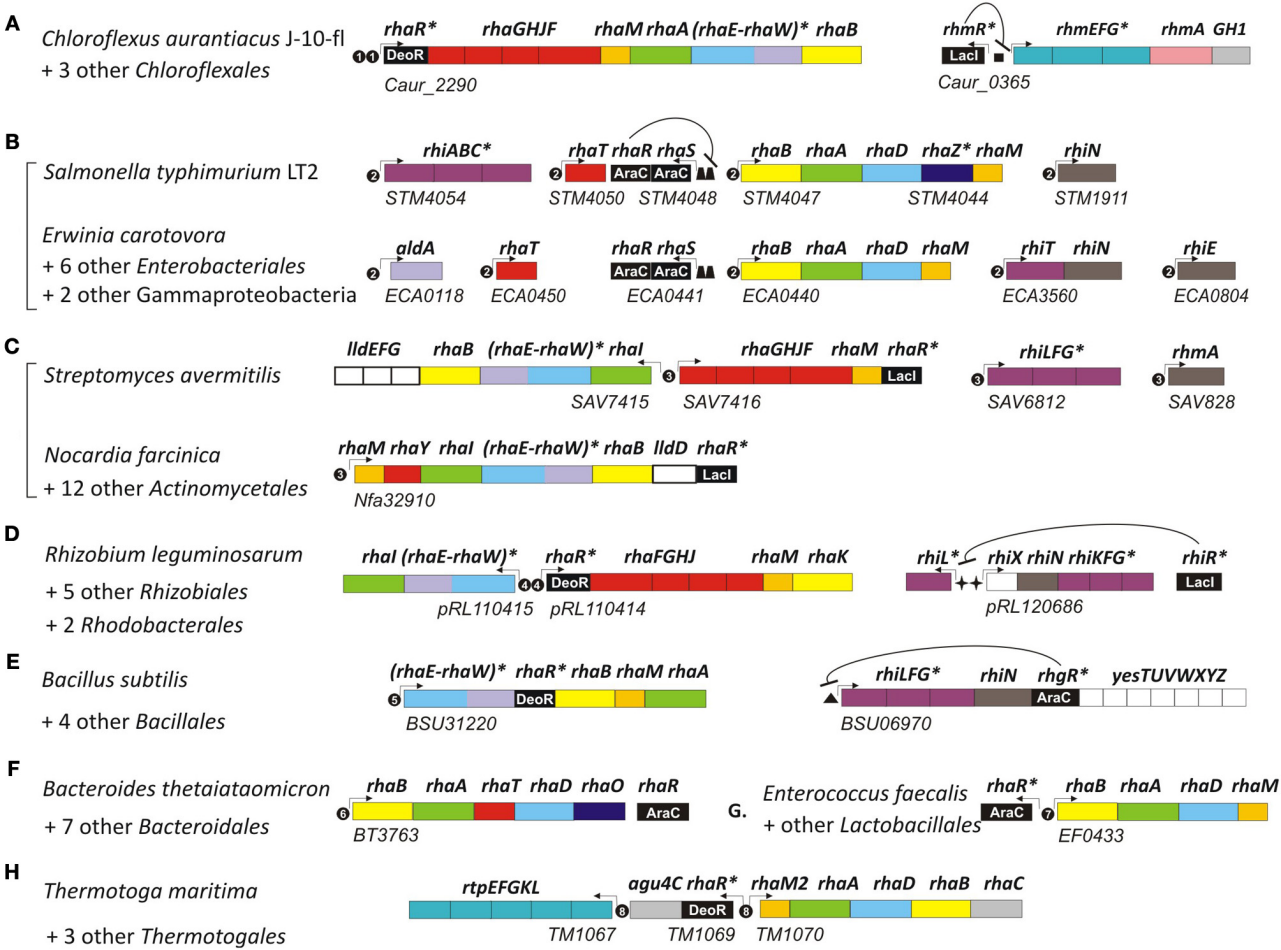
**Genomic context of L-rhamnose catabolic genes and regulons in bacteria from seven diverse taxonomic lineages.** Genes (shown by rectangles) with the same functional roles are marked in matching colors. Genes encoding the novel bifunctional enzyme RhaEW are in parenthesis. Tentatively predicted functional roles are marked by asterisks. Transcriptional regulators are in black with the corresponding protein family name indicated by white text. Potential promoters are indicated by small arrows. Candidate regulator binding sites are shown by black circles with number corresponding to the DNA binding motifs in Figure 3, as well as by squares, trapezoids, stars, and triangles. Genomic locus tags of the first gene are indicated below each putative operon. Bacterial lineages: **(A)** *Chloroflexales*; **(B)** *Enterobacteriales*; **(C)** *Actinomycetales*; **(D)** *Rhizobiales*; **(E)** *Bacillales*; **(F)** *Bacteroidales*; **(G)** *Lactobacillales*; **(H)** *Thermotogales*.

**Figure 3:**
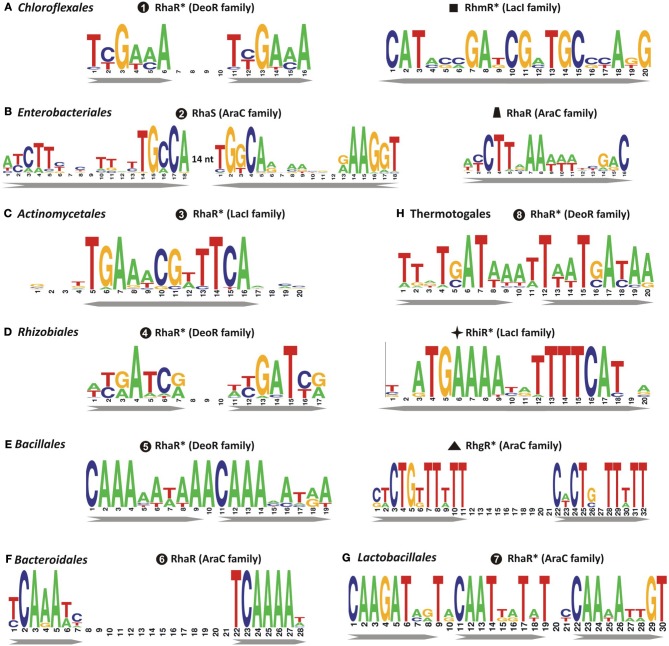
**Consensus sequence logos for predicted DNA binding sites of transcriptional regulators of L-rhamnose catabolism in diverse bacterial lineages.** Sequence logos representing the consensus binding site motifs were built using all candidate sites in each microbial lineage that are accessible in the RegPrecise database. Previously uncharacterized regulators with tentatively predicted DNA motifs are marked by asterisks. Bacterial lineages: **(A)** *Chloroflexales*; **(B)** *Enterobacteriales*; **(C)** *Actinomycetales*; **(D)** *Rhizobiales*; **(E)** *Bacillales*; **(F)** *Bacteroidales*; **(G)** *Lactobacillales*; **(H)** *Thermotogales*.

The transcriptional regulator RhaS in *E. coli* belongs to the AraC protein family and controls the L-Rha transporter *rhaT* and the catabolic operon *rhaBADU* (Egan and Schleif, 1993; Via et al., 1996). Orthologs of *rhaS* and these catabolic genes for L-Rha utilization are present in other *Enterobacteriales*, as well as in *Tolumonas* and *Mannheimia* spp. RhaS in *E. chrysanthemi* was additionally shown to regulate the *rhiTN* operon involved in the uptake and catabolism of rhamnogalacturonides, L-rhamnose containing oligosaccharides (Hugouvieux-Cotte-Pattat, 2004). The analysis of upstream regions of RhaS-controlled genes and their orthologs in γ-proteobacteria resulted in identification of the putative RhaS-binding motif, which was used for identification of additional RhaS targets in the analyzed genomes (Figures 2B, 3B).

Analysis of other taxonomic groups outside the γ-proteobacteria identified previously uncharacterized members of the LacI, DeoR, and AraC families as alternative transcriptional regulators of the L-rhamnose catabolic pathways (Figure 2). To infer novel L-Rha regulons in each taxonomic group, we applied the comparative genomics approach that combines identification of candidate regulator-binding sites with cross-genomic comparison of regulons. The upstream regions of L-Rha utilization genes in each group of genomes containing an orthologous TF was analyzed using a motif-recognition program to identify conserved TF-binding DNA motifs (Figure 3). The deduced palindromic DNA motifs of novel LacI-family regulators are characteristic of DNA-binding sites of LacI family regulators. The predicted binding motifs of DeoR-family RhaR regulators in four distinct taxonomic groups are characterized by unique sequences; however, each of them has a similar structure that includes two imperfect direct repeats with a periodicity of 10–11 bp. Novel AraC-family regulators of L-Rha metabolism in the *Bacillales*, *Bacteroides*, and *Enterococcus* groups also are characterized by unique DNA motifs with a common structure of a direct repeat with 21-bp periodicity. Among this large set of predicted L-Rha catabolic regulators, only two transcriptional factors, an AraC-type activator in *B. thetaiotaomicron* and a DeoR-type repressor in *R. leguminosarum*, have been shown experimentally to mediate the transcriptional control of L-Rha utilization genes in the previous studies (Richardson et al., 2004; Patel et al., 2008), although specific DNA operator motifs of these two regulators were not reported before.

A detailed description of the reconstructed L-Rha catabolic regulons is available in the RegPrecise database within the collection of regulons involved in L-Rha utilization (Novichkov et al., 2013). Overall, most of these TF regulons are local and control from one to several target operons per genome (Figure 2). In the *Bacillales*, RhaR and RhgR control genes involved in the utilization of L-rhamnose and rhamnogalacturonan, respectively (Leyn et al., 2013). In the *Thermotogales*, the DeoR-family RhaR regulator co-regulates genes involved in the utilization of L-Rha mono- and oligosaccharides (Rodionov et al., 2013). In the *Rhizobiales*, RhaR from the DeoR family negatively controls the L-Rha catabolic operon (Richardson et al., 2004), whereas RhiR from the LacI family is predicted to regulate the rhamnogalacturonide utilization gene cluster (named *rhi*). An orthologous LacI-family regulator controls the similar *rhi* gene locus in *Erwinia* spp. In the *Actinomycetales*, a novel LacI-type regulator (termed RhaR) co-regulates genes involved in the uptake and catabolism of L-Rha and L-Rha-containing oligosaccharides. In the *Chloroflexales*, two unique TFs control L-Rha metabolism—the DeoR-family regulator RhaR controls the L-Rha utilization operons in both *Chloroflexus* and *Roseiflexus* spp., while the LacI-family regulator RhmR controls the *rhm* operon involved in the L-Rha oligosaccharide utilization in *C. aurantiacus*.

In summary, at least seven non-orthologous types of TFs appear to regulate the L-rhamnose utilization (*rha*) genes in diverse bacterial lineages. Uptake and catabolism of L-Rha-containing oligosaccharides is either co-regulated with *rha* genes by the same TFs (e.g., RhaRs in *Actinomycetales* and *Thermotogales*; RhaS in *Enterobacteriales*), or is under control of other specialized TFs (RhgR in *Bacilales*, RhiR in *Rhizobiales*, and *Erwinia*, RhmR in *Chloroflexus*). In the third part of this study, we experimentally validated the predicted DNA binding sites of RhaR regulator in *C. aurantiacus*.

#### L-rhamnose catabolic pathways

Analysis of L-Rha regulons revealed various sets of genes that are presumably involved in the L-rhamnose utilization subsystem (Table S1 in Supplementary Material). By analyzing protein similarities and genomic contexts for these genes, we inferred their potential functional roles and reconstructed the pathways (Figure 1). All four enzymatic steps of the reconstructed catabolic pathways occur in many alternative forms. The most conserved enzyme in the L-Rha subsystem is the L-rhamnulose kinase RhaB, which is substituted by a non-orthologous kinase from the same protein family in γ-proteobacteria (Rodionova et al., 2012b). Two alternative types of L-rhamnulose isomerase (RhaA and RhaI) are almost equally distributed among the studied genomes. All analyzed lineages except the *Bacilalles* possess L-rhamnulose isomerases of a single type. Among the *Bacillales*, all studied genomes have the RhaA isomerase, whereas only *B. licheniformis* has the non-orthologous isozyme RhaI.

The canonical form of L-rhamnulose-1-P aldolase (RhaD) was found in γ-proteobacteria, *Bacteroidales*, *Thermotogales*, and *Lactobacillales*. Instead of RhaD, the L-Rha catabolic gene clusters in *Actinomycetales*, α-proteobacteria, *Bacillales*, and *Chloroflexales* contain a chimeric gene encoding a two-domain protein (e.g., *yuxG* in *Bacillus subtilis*). The uncharacterized protein YuxG and its orthologs have an N-terminal class II aldolase domain (PF00596 protein family in PFAM) fused to a C-terminal short-chain dehydrogenase domain (PF00106). We used DELTA_BLAST to search for distant homologues of YuxG among proteins with experimentally determined functions. The N-terminal domain of YuxG (named RhaE) is distantly homologous to three *E. coli* enzymes, L-ribulose-5-phosphate epimerase (15% identity, *E*-value 1e^−39^), L-fuculose-1-phosphate aldolase (11% identity, *E*-value 4e^−36^), and the canonical RhaD enzyme (14% identity, *E*-value 1e^−25^). These relationships suggest that it represents a non-orthologous substitution of aldolase RhaD. The C-terminal domain of YuxG (named RhaW) is homologous to various NADH-and NADPH-dependent sugar dehydrogenases including sorbose dehydrogenase from fungi (29% identity, *E*-value 3e^−14^), 2,3-butanediol dehydrogenase from *Corynebacterium glutamicum* (25% identity, *E*-value 4e^−10^), and sorbitol-6-phosphate dehydrogenase from *E. coli* (22% identity, *E*-value 9e^−09^). The phylogenetic occurrence profile suggests that RhaW may encode the missing L-lactaldehyde dehydrogenease/reductase. Thus, the bifunctional protein RhaEW is tentatively predicted to catalyze the two final reactions in the L-Rha catabolic pathway (Figure 1).

Downstream enzymes for utilization of L-lactaldehyde varied the most among the analyzed species. Reconstruction of the RhaS regulon in γ-proteobacteria identified various genes that are likely involved in utilization of L-lactaldehyde. The rhamnose operons in *S. typhimurium* and five other species include an additional gene (named *rhaZ*) that encodes a hypothetical iron-containing alcohol dehydrogenase (PF00465). *E. carotovora* has a single RhaS-regulated gene *aldA* encoding alcohol dehydrogenase from another protein family (PF00171). In contrast, the RhaS regulons in *E. chrysanthemi* and *Mannheimia* spp. include the L-lactaldehyde reductase *rhaO*, whereas *aldA* and *rhaZ* are absent from their genomes. These observations suggest that γ-proteobacteria use three different enzymes and two different pathways for the final stage of the L-rhamnose pathway (Figure 1).

In summary, the subsystem reconstruction and genome context analyses allowed us to predict the following novel candidate genes: L-rhamnulose-1-P aldolase (RhaE) and two variants of L-lactaldehyde utilizing enzymes (RhaW and RhaZ) in diverse bacterial genomes. In the second part of this study, we experimentally validated the predicted functions of RhaEW from *B. subtilis* and*C. aurantiacus* and RhaZ from *S. typhimurium*.

#### L-rhamnose transporters and upstream hydrolytic pathways

Uptake of L-Rha in *E. coli* is mediated by the L-Rha–proton symport protein, RhaT (Baldoma et al., 1990) that belongs to the Drug/Metabolite Transporter (DMT) superfamily. An orthologous L-Rha transporter was found in the genome context of L-Rha utilization genes/regulons in other γ-proteobacteria and in the *Bacteroidales* (Table S1 in Supplementary Material). Another L-Rha transporter belonging to the ABC superfamily, RhaSTPQ (designated RhaFGHJ here) was described in *R. leguminosarum* (Richardson et al., 2004). In this study, we identified orthologs within the L-Rha operons/regulons in all other α-proteobacteria, as well as in several genomes from the *Chloroflexales*, *Actinomycetales*, and *Enterobacteriales* orders. A different putative L-Rha transporter (termed RhaY), which belongs to the Sugar Porter (SP) family of the Major Facilitator Superfamily (MFS), was identified in certain *Bacillales* and *Actinomycetales* genomes. This functional assignment is supported by the conserved co-localization on the chromosome (in *Mycobacterium*/*Nocardia* spp.) and by predicted co-regulation (via upstream RhaR-binding site in *Saccharopolyspora erythraea*) with other *rha* genes.

The predicted L-Rha regulons in many bacteria include several glycoside hydrolases and transport systems involved in the uptake of L-Rha-containing oligosaccharides in the cytoplasm and their consequent degradation to form L-Rha monosaccharides. The RhaS-activated operon *rhiTN* is involved in the uptake and hydrolysis of oligosaccharides produced during rhamnogalacturonan catabolism in the plant-pathogenic species from the order *Enterobacteriales* (Hugouvieux-Cotte-Pattat, 2004). Another enterobacterium, *S. typhimurium*, possesses a different RhaS-regulated transport system (named *rhiABC*), which is similar to the C4-dicarboxylate transport system Dcu (Figure 2). Based on the gene occurrence pattern and candidate co-regulation, *rhiABC* is tentatively predicted to encode an alternative transporter for rhamnogalacturonides, which replaces RhiT in *S. typhimurium*. A different transport system from the ABC family (named *rhiLFG*) and putative α-L-rhamnosidases (*ramA*, *rhmA*) were detected within the RhaR regulons in several *Actinomycetales*. In the *Bacillales* and *Rhizobiales* groups, as well as in the *Ewrinia* and *Chloroflexus* spp., homologous ABC transporters and rhamnohydrolases are co-regulated with several novel lineage-specific transcriptional regulons, RhgR, RhiR, and RhmR, respectively.

In summary, the comparative genomics analysis of L-Rha catabolic subsystem in bacteria revealed extensive variation for the components of transport machinery. L-Rha transport systems belong to at least three protein families. In addition to L-Rha transporters, many L-Rha-utilizing bacteria possess systems for active uptake of L-Rha containing oligosaccharides.

### Experimental validation of novel rhamnose catabolic enzymes

#### Novel aldolase/dehydrogenase RhaEW

To provide biochemical evidence for the novel bifunctional aldolase/dehydrogenease enzyme involved in L-Rha catabolism, the recombinant protein RhaEW from *C. aurantiacus* (termed *Ca*_RhaEW) was overexpressed in *E. coli* with the N-terminal His6 tag, purified using Ni-NTA affinity chromatography, and characterized *in vitro* by a coupled enzymatic assay using spectrophotometry and GC-MS.

**Figure 4:**
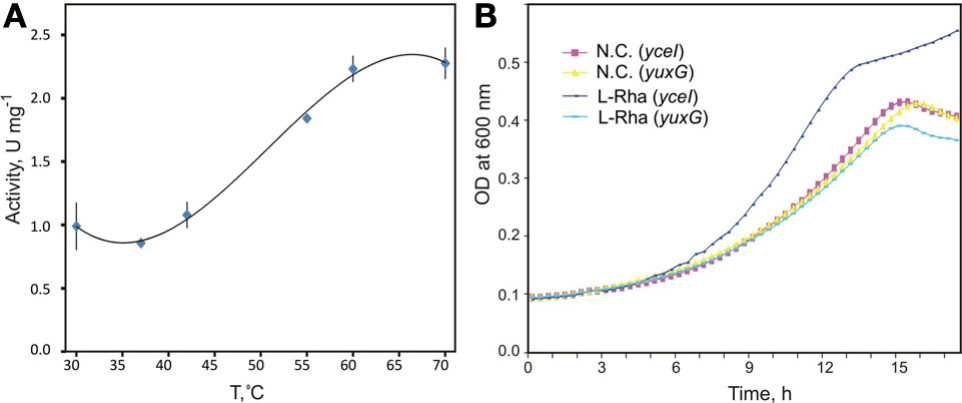
**Biochemical and physiological characterization of novel aldolase/dehydrogenase RhaEW. (A)** Temperature dependence of enzymatic activity of recombinant RhaEW protein from *C. aurantiacus* determined by a coupling colorimetric assay of the NAD-dependent RhaW dehydrogenase activity. **(B)** Growth studies of *B. subtilis* knockout mutants for *yuxG* (*rhaEW*) and *yceI* (*niaP* gene used as a control) grown in defined medium in the presence of L-rhamnose, D-glucose, and no additional carbon source (N.C.). Growth studies were conducted in triplicate.

Bioinformatics analysis suggested that RhaEW is a bifunctional enzyme catalyzing two sequential activities, L-rhamnulose-1-P aldolase and L-lactaldehyde dehydrogenase (Figure 1). We assayed the biochemical activity of the recombinant *Ca*_RhaEW protein by monitoring the conversion of NAD^+^ to NADH at 340 nm as a result of predicted L-lactaldehyde dehydrogenase reaction. The peak of *Ca*_RhaEW activity (*V*_*max*_ 2.9 U mg Major Facilitator Superfamily^−1^) was observed at 60–70°C (Figure 4A), which is in agreement with the optimal temperature range for the growth for *C. aurantiacus* (Hanada and Pierson, 2006). Additionally, we tested the possibility that *Ca*_RhaEW acts as an aldolase/reductase by supplying NADH rather than NAD^+^ in the reaction; no activity was seen under these conditions (data not shown). Thus, *Ca*_RhaEW acts *in vitro* to convert L-rhamnulose-1-P to L-lactate and DHAP, which is consistent with the prediction made through comparative genomics analyses.

The formation of *Ca*_RhaEW reaction products was directly confirmed by GC-MS profiling of reaction mixtures obtained by overnight incubation of L-Rha with the *Ca*_RhaEW protein taken alone or in combination with the upstream catabolic enzymes. While incubation of L-Rha with *Ca*_RhaEW alone did not produce any new peaks on the chromatogram, the addition to the mixture of the *Ec*_RhaA and *Tm*_RhaB proteins led to a decrease of two peaks corresponding to L-Rha (retention times 9.28 and 9.39 min) and the appearance of a series of novel peaks (Figure S1 in Supplementary Material). By comparison with standards and the analysis of electron ionization mass spectra (*m/z* 299), the first two peaks with retention times 9.27 and 9.37 min were attributed to DHAP, whereas the peak at retention time 7.75 min was assigned as lactate. Additional peaks appearing in the coupled enzymatic assay were attributed to the upstream intermediates of the L-Rha catabolic pathway, L-rhamnulose (retention times 8.85 and 8.92 min) and L-rhamnulose-1-P (13.04 min). The moderate consumption of L-Rha observed when only *Ec*_RhaA and *Tm*_RhaB enzymes were added increased substantially after addition of *Ca*_RhaEW to the reaction mixture. Finally, neither DHAP nor lactate was detected in the reaction mixture after exclusion of NAD^+^ which is an essential cofactor of L-lactaldehyde dehydrogenase. These results suggest that the activity of the L-lactaldehyde dehydrogenase domain RhaW is essential for the L-rhamnulose-1-P aldolase activity of the second domain in this bifunctional enzyme.

In order to test the hypothesis that RhaEW from *B. subtilis* functions in the catabolism of L-Rha *in vivo*, we performed growth experiments in defined medium for two mutant *B. subtilis* strains. One strain carried a knockout mutation in the gene *yuxG* (*rhaEW*), whereas the second strain carried an intact version of *yuxG* but had a knockout mutation in an unrelated gene, *yceI* (encoding a niacin transporter), to serve as an isogenic control. We expected that the growth of the *B. subtilis yuxG* mutant strain would not be stimulated by the addition of L-Rha as a carbon source when compared to the *yceI* mutant strain. The results clearly demonstrate that the *B. subtilis yuxG* knockout mutant is non-responsive to added L-Rha when compared to the *yceI* knockout strain and to both strains grown in the absence of an additional carbon source (Figure 4B). These data confirm that RhaEW is required for L-Rha utilization in *B. subtilis*. The *B. subtilis* RhaEW protein (*Bs*_RhaEW) was cloned, purified, and tested by the same coupled enzymatic assay as described above for *Ca*_RhaEW. The *Bs*_RhaEW protein showed weak, but reproducible activity, measured at 0.0127 ± 0.001 μmol mg protein^−1^ min^−1^ at 25°C. Controls removing starting substrate (L-Rha), *Bs*_RhaEW, or *Ec*_RhaB (effectively removing rhamnulose-1-P) from the reaction yielded no measurable activity (Figure S2A in Supplementary Materials).

#### Rhaz functions as a L-lactaldehyde reductase in vitro

We used the reconstituted L-Rha catabolic pathway to test the prediction that *Salmonella* spp. harbor a novel L-lactaldehyde dehydrogenase, distinct from that of *E. coli* and shared among a subgroup of the γ-proteobacteria. We cloned, overexpressed and purified the recombinant proteins *St*_RhaD (predicted aldolase) and *St*_RhaZ (predicted novel dehydrogenase) from *S. typhimurium* to complete the *in vitro* pathway (Figure 1). *St*_RhaD is 99% identical at the amino acid level to *E. coli* RhaD, for which an aldolase function has been demonstrated (Schwartz et al., 1974). To ensure that *St*_RhaD acts as an aldolase in the L-Rha catabolism, we performed two control assays to confirm the production of DHAP and L-lactaldehyde. To test for the production of DHAP, we used purified glycerol-3-P dehydrogenase (GPDH) (Sigma) in an assay containing *Ec*_RhaA, *Tm*_RhaB, and *St*_RhaD. If *St*_RhaD acts as a L-rhamnulose-1-P aldolase, then the DHAP produced would be converted to glycerol-3-P by GPDH with the oxidation of NADH to NAD^+^ monitored as a decrease in absorbance at 340 nm. Likewise, it was expected that if *St*_RhaD produced L-lactaldehyde, then the known *E. coli* L-lactaldehyde dehydrogenase, AldA, should be active in a reaction containing all three L-Rha catabolic enzymes, producing L-lactate, and converting NAD^+^ to NADH. The results of both controls confirmed the activity of *St*_RhaD as a L-rhamnulose-1-P aldolase (data not shown), making possible to test the prediction for *St*_RhaZ. The purified *St*_RhaZ protein was included in an assay containing *Ec*_RhaA, *Tm*_RhaB, and *St*_RhaD, using NAD^+^ as a cofactor. This reaction mixture should lead to the conversion of L-lactaldehyde to L-lactate (as with the *E. coli* AldA enzyme). Under these conditions, *St*_RhaZ did not show activity as a L-lactaldehyde dehydrogenase. In order to assess the alternative fate for L-lactaldehyde, which is conversion to L-1,2-propanediol, we repeated the assay under identical conditions with the exception of supplying NADH as the cofactor. *St*_RhaZ was active under these conditions (Figure 2B in Supplementary Materials), converting L-lactaldehyde to L-1,2-propanediol with a specific activity of 0.13 ± 0.02 μmol mg protein^−1^ min^−1^. This indicates that the function of RhaZ is a L-lactaldehyde reductase, rather than a L-lactaldehyde dehydrogenase.

### Experimental validation of rhamnose utilization and regulon in chloroflexus aurantiacus

The anoxygenic phototroph *C. aurantiacus* can grow heterotrophically using various organic compounds under either oxic conditions or anoxic conditions in light (Hanada and Pierson, 2006). However, the ability of *C. aurantiacus* and other species from the order *Chloroflexales* to utilize L-Rha has not been previously investigated. In *C. aurantiacus*, the L-Rha utilization genes are organized into a nine-gene *rha* operon, which is predicted to be transcriptionally controlled by a novel DeoR-family regulator RhaR (Figure 2). An additional gene, termed *rhmA*, encoding a potential α-L-rhamnosidase (*Caur_0361*) is potentially involved in the utilization of L-Rha oligosaccharides by *C. aurantiacus*. A novel LacI-family transcription factor, termed RhmR, potentially regulates the RhmA-encoding operon, which also encodes a potential transport system for uptake of L-Rha-containing oligosaccharides, termed RhmEFG (Figure 2). In contrast to the L-Rha utilization operon, which has orthologs in all sequenced genomes of *Chloroflexus* and *Roseiflexus* spp., the *rhmR/A* gene locus is only conserved in the closely-related *Chloroflexus* spp. strain, Y-400-fl, but is absent in the other *Chloroflexales*. We assessed the L-Rha utilization and regulon in *C. aurantiacus* by a combination of *in vivo* and *in vitro* experimental approaches.

To validate L-Rha-specific induction of the predicted L-Rha utilization genes *in vivo*, we performed RT-PCR with specific primers designed for three *rha* operon genes, *rhaR*, *rhaF*, and *rhaB*. Total RNA was isolated from *C. aurantiacus* grown in media containing YE or pyruvate, with and without addition of L-Rha. All three genes demonstrated elevated transcript levels in the cells grown on either YE or pyruvate media supplied with L-Rha compared to that of the cells grown in the absence of L-Rha (Figure S3 in Supplementary Materials). In addition to the *rha* operon genes, *rhmA* transcription was also highly elevated in pyruvate-grown cells supplied with L-Rha. These results confirm that the *rha* and *rhm* operons, that are predicted to be controlled by RhaR and RhmR transcription factors, respectively, are transcriptionally induced by L-Rha. Additionally, the L-Rha grown culture samples of *C. aurantiacus* were analyzed by HPLC to monitor the L-Rha consumption from the culture fluids. The results confirm a high rate of L-Rha consumption in the samples (Figure S3 in Supplementary Materials), thus confirming that the L-Rha uptake and utilization system is functional *in vivo*.

**Figure 5:**
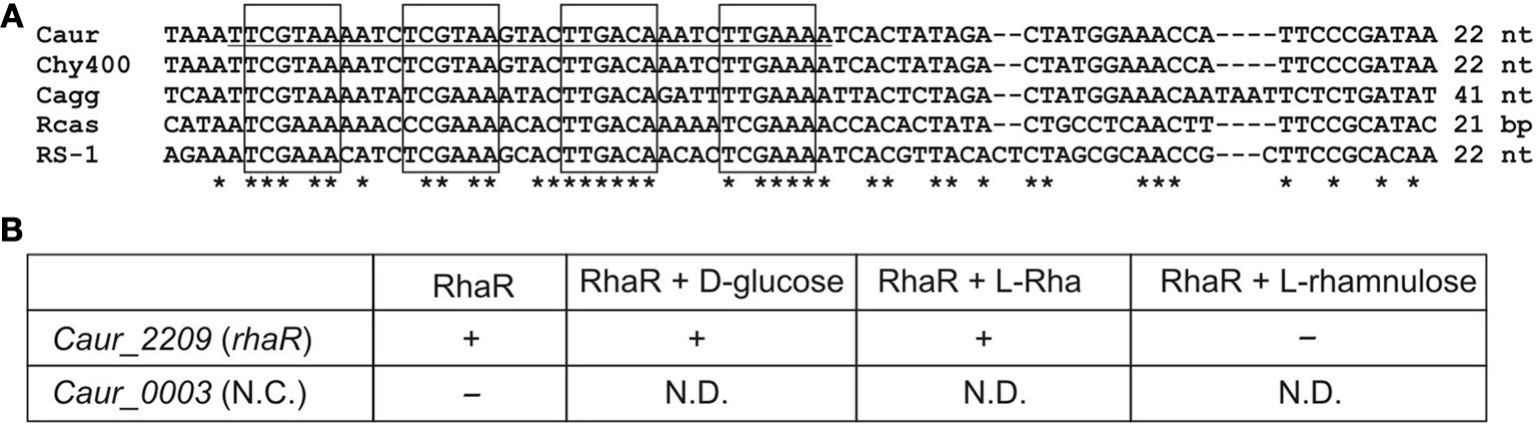
**Experimental validation of RhaR regulon in *C. aurantiacus*. (A)** Conservation of predicted RhaR binding sites (boxed) identified in the promoter regions of *rha* operons in the *C. aurantiacus* J-10-fl (Caur), *C*. sp. Y-400-fl (Chy400), *C. aggregans* DSM 9485 (Cagg), *Roseiflexus* sp. RS-1 (RS-1), and *R. castenholzii* DSM 13941 (Rcas). Distance to a start codon of *rhaR* is indicated. A 38-bp fragment from *C. aurantiacus* used for DNA binding assays is underlined. **(B)** Summary of the EMSA experiments assessing the potential interaction between the recombinant RhaR protein and its predicted DNA motif at the *Caur_2209* (*rhaR*) gene. The disappearance of unbound DNA band (shown by “+”) was observed upon the addition of increasing concentrations of RhaR protein (0.25–1 μ M). Addition of 2 mM of L-rhamnose or D-glucose to the reaction mixture containing 1 μ M of RhaR did not change this pattern, whereas addition of 2 mM of L-rhamnulose led to re-appearance of the unbound DNA band (shown by “–”). As a negative control, incubation of RhaR protein (0.5 μ M) with upstream DNA fragment of *Caur_0003* did not reveal the disappearance of unbound DNA band (shown by “–”). The EMSA gel pictures are presented in Figure S4 in Supplementary Material. Asterisks indicate the conserved nucleotides in the multiple alignment.

The interaction of the predicted RhaR regulator with the *Caur_2209* (*rhaR*) upstream DNA fragment containing candidate RhaR-binding sites in *C. aurantiacus*, and the influence of potential sugar effectors on protein-DNA interaction were assessed *in vitro* by EMSA (Figure 5). The synthetic 38-bp DNA region containing a tandem repeat of four individual RhaR sites (a consensus sequence TCGAAA) was incubated with increasing concentrations of the purified recombinant RhaR protein. The incubation was performed at 50°C, which is close to the optimal growth temperature of 55°C for *C. aurantiacus*. The EMSA results (Figure S4 in Supplementary Material) are consistent with the *in silico* predicted DNA operator region of RhaR. The addition of D-glucose and L-Rha had no effect on RhaR-DNA interaction, whereas L-rhamnulose abolished the specific DNA-binding ability of RhaR. The obtained results suggest that the RhaR repressor binds to the operator region at the *rha* operon in the absence of a sugar inducer, and that L-rhamnulose serves as a negative regulator for RhaR in *C. aurantiacus*.

## Discussion

L-Rha is the most common deoxy-hexose sugar in nature. In plants, it is a component of many glycosides and polysaccharides such as pectins and hemicelluloses (Peng et al., 2012). Among bacteria, L-Rha is found in the cell wall and as a part of the glycosylated carotenoids (Takaichi and Mochimaru, 2007; Takaichi et al., 2010). Utilization of L-Rha and rhamnose-containing polysaccharides has previously been studied in several free-living and plant pathogenic microbial species from the phylum *Proteobacteria*, including members of the genera *Escherichia*, *Erwinia*, *Rhizobium*, *Azotobacter*, and *Sphingomonas*. Due to significant variations in sugar catabolic pathways in bacteria, the projection of this knowledge to the genomes of more distant species, including many species important for prospective bioenergy applications, is a challenging problem (Rodionov et al., 2010, 2013). In this study, we used comparative genomics to reconstruct novel variants of catabolic pathways and novel transcriptional regulons for L-Rha utilization in the genomes of bacteria from ten taxonomic groups.

Using bioinformatics analyses of L-Rha utilization genes, we identified twelve groups of rhamnose-related transcriptional regulators from different protein families, AraC, DeoR, and LacI, and proposed binding site motifs for these regulators within tentatively reconstructed regulons (Figure S5 in Supplementary Material). Prior to this study, only four types of bacterial transcriptional regulators related to L-Rha metabolism had been identified. The AraC family includes at least five groups of non-orthologous regulators of L-Rha metabolism. These regulators have unique DNA motifs with a tandem repeat symmetry. Activators from three AraC groups have been characterized previously: RhaR and RhaS from *E. coli* and *Erwinia* spp., with previously known DNA motifs, and RhaR from *Bacteroides*, with previously unknown DNA motif. The DeoR family includes at least four non-orthologous groups of RhaR regulators that are characterized by distinct DNA motifs with a tandem repeat symmetry. Among them, only RhaR in *Rhizobium* spp. was described previously (Richardson et al., 2004); however, its DNA binding motif was not known before this study. All LacI-family regulons of L-Rha utilization genes were analyzed for the first time in this study. They are characterized by 20-bp palindromic DNA motifs of four different consensus sequences. In summary, the results of this comparative genomics study demonstrate significant variability in the design and composition of transcriptional regulons for L-Rha metabolism in bacteria. This study has very significantly increased our knowledge about types and operator sequences for transcriptional regulators for L-Rha utilization.

Based on genomic context analyses of the reconstructed regulons, we have identified several novel enzymes and transporters involved in L-Rha utilization (Figure 1). A novel enzyme with two domains, termed RhaEW, encoded by the *yuxG* gene in *B. subtilis* and its orthologs in other bacterial lineages, was found to catalyze the last two steps in the catabolism of L-Rha, namely cleavage of L-rhamnulose-1-P to produce DHAP and L-lactaldehyde and oxidation of L-lactaldehyde to L-lactate. Thus, the RhaE domain functions as a non-orthologous substitute for the classical RhaD aldolase, whereas the function of the RhaW domain is analogous to the aldehyde dehydrogenase AldA from *E. coli*. A novel L-lactaldehyde reductase involved in L-Rha catabolism, termed RhaZ, that is not homologous to previously characterized RhaO/FucO, was identified in many γ-proteobacteria. Both functional predictions were experimentally validated *in vitro* by enzymatic assays with the purified recombinant proteins from *C. aurantiacus* and *B. subtilis* (for RhaEW), and *S. typhimurium* (for RhaZ). The function of RhaEW in L-Rha utilization *in vivo* was also confirmed by genetic techniques in *B. subtilis*. Interestingly, genes encoding L-lactate dehydrogenases (*lldD*, *lldEFG*) belong to the reconstructed RhaR regulons in certain genomes of the *Actinomycetales* and *Rhodobacterales* that encode RhaEW. Thus, the L-Rha utilization pathways in these species are probably extended to produce pyruvate as one of the final products.

Orthologs of the novel aldolase/dehydrogenase RhaEW are broadly distributed among diverse bacterial phyla including Proteobacteria (α-subdivision), Actinobacteria, Chloroflexi, Bacteroidetes, and Firmicutes (*Bacillales*), in which they are always encoded within the *rha* gene loci (Figure S6 in Supplementary Material). The L-rhamnulose-1-P aldolase domain in RhaE is distantly homologous to class II aldolases including the analogs enzyme, RhaD, and the L-fuculose-1-P aldolase, FucA, from *E. coli*. The tertiary structures and catalytic mechanisms for these enzymes have been determined (Dreyer and Schulz, 1996; Grueninger and Schulz, 2008). We aligned the amino acid sequences of all three enzymes using the multiple protein sequence and structure alignment server PROMALS3D (Pei et al., 2008) (Figure S7 in Supplementary Material). Class II aldolases are zinc-dependent enzymes, in which the metal ion is used for enolate stabilization during catalysis. In RhaD, the Zn^2+^ ion is chelated by three histidines, His^141^, His^143^, and His^212^, which are conserved in all RhaE proteins. An Asp residue in RhaE replaces the catalytically important Glu^117^ in RhaD, which performs the nucleophilic attack of the C3 atom of DHAP. This conservative substitution suggests that this Asp may play the similar role in RhaE. The Gly^28^, Asn^29^, and Gly^44^ residues that are involved in phosphate binding in FucA (Dreyer and Schulz, 1996) are conserved in both RhaD and RhaE enzymes. Conservation of the catalytically important amino acids in both types of L-rhamnulose-1-P aldolases suggests similar position of the active site and catalytic mechanism.

In summary, the phosphorylated catabolic pathway for L-Rha contains a large number of alternative enzymes including RhaI/RhaA, RhaB/RhaK, RhaD/RhaE, RhaO/RhaZ, and RhaW/AldA (Figure 1) and is widely-distributed among diverse bacterial phyla. An alternative pathway for the nonphosphorylated L-Rha catabolism that utilizes a unique subset of catabolic enzymes was found only in a small number of proteobacteria (Table S1 in Supplementary Material). In addition to numerous variations among enzymes and transcriptional regulators associated with the L-Rha catabolic pathway, a similarly high level of variations and non-orthologous displacements is observed for the components of transport machinery. The L-Rha permease, RhaT, which is characteristic of members of the *Enterobacteriales* and *Bacteroidales*, appears to be functionally replaced by either a permease from a different family in some *Actinomycetales* and *Bacillales* or an ABC cassette in α-proteobacteria and *Chloroflexales*. In other genomes, no candidate transporter specific for L-Rha was detected; however, the reconstructed L-Rha pathways and regulons in these species include transport systems and hydrolytic enzymes for L-Rha oligosaccharides (e.g., rhamnogalacturonides). Some of the latter species are known to grow on L-Rha, such as *B. subtilis* (this study) and *T. maritima* (Rodionov et al., 2013), thus we propose that the predicted L-Rha oligosaccharide transporters in these species are also capable of L-Rha uptake.

Previous studies of L-Rha catabolism in *E. coli* and *Salmonella*, revealed a differential fate for L-Rha under aerobic and anaerobic conditions in *E. coli*, but not in *Salmonella* (Baldoma et al., 1988; Obradors et al., 1988). *E. coli* oxidizes L-lactaldehyde to L-lactate via the activity of AldA under aerobic conditions and reduces L-lactaldehyde to L-1,2-propanediol via the activity of FucO under anaerobic conditions (Figure 1). In contrast, *Salmonella* produces L-1,2-propanediol under both aerobic and anaerobic conditions when metabolizing L-Rha. The identification of *Salmonella* RhaZ as an L-lactaldehyde reductase is consistent with these observations. *Salmonella* produce 1:1 molar equivalents of L-1,2-propanediol from the catabolism of L-Rha under both aerobic and anaerobic conditions, with growth yields higher than *E. coli* under anaerobic conditions (Baldoma et al., 1988). The production of L-1,2-propanediol through renewable, biological methods is of high importance given the current chemical based processes of production and the high use of L-1,2-propanediol in many commercial products (Cameron et al., 1998). There are several examples of recent bioengineering strategies to improve L-1,2-propanediol production in *E. coli* (Clomburg and Gonzalez, 2011), cyanobacteria (Li and Liao, 2013), and *Saccharomyces* (Jung et al., 2011) in which each strategy uses glycerol as a starting substrate. The observation of differential fates for L-Rha in *E. coli* and *Salmonella*, the identification of the activity of RhaZ, putative transport systems for rhamnogalacturonides, and predicted regulatory mechanisms in *Salmonella* raise possibilities for exploring alternative biological production strategies of the commercially important L-1,2-propanediol from L-Rha containing substrates, though L-Rha, itself, remains an expensive substrate (Cameron et al., 1998).

*C. aurantiacus* and other filamentous anoxygenic phototrophic bacteria from the *Chloroflexaceae* family were commonly found in the upper layers of microbial mats in hot springs (50–62°C), with cyanobacteria growing together with chloroflexi. Although *Chloroflexus* spp. can grow heterotrophically on various organic carbon sources, their sugar utilization pathways have remained largely unknown before this work. Here, we identified and characterized a novel variant of the L-Rha catabolic pathway in *C. aurantiacus*, which includes the L-Rha isomerase RhaA, kinase RhaB, and a novel bifunctional enzyme, RhaEW, that catalyzes the last two steps of the pathway. *C. aurantiacus* transcribed genes for L-Rha utilization when L-Rha was present in the growth medium and consumed L-Rha from the medium. The ecophysiological importance of the L-Rha utilization pathway in members of the *Chloroflexales* is yet to be elucidated. One possibility is that cyanobacteria commonly co-occurring with chloroflexi in hot springs microbial mats may provide them L-Rha. In such microbial mats, cyanobacteria are primary producers that are thought to cross-feed low-molecular-weight organic compounds (e.g., lactate, acetate, glycolate) to members of the *Chloroflexales* (van der Meer et al., 2003, 2007). There are several potential sources of L-Rha in cyanobacteria including lipopolysaccharides in the outer membrane (Buttke and Ingram, 1975) and glycosylated carotenoids in the cytoplasmic and outer membrane that protect the cell against photooxidative damage (Takaichi and Mochimaru, 2007; Graham and Bryant, 2009). The exact source of L-Rha from a primary producer and its significance for possible metabolite exchange in the mat community requires further investigation.

### Conflict of interest statement

The authors declare that the research was conducted in the absence of any commercial or financial relationships that could be construed as a potential conflict of interest.
