# Supplementary material for: Comparative genomics and functional analysis of rhamnose catabolic pathways and regulons in bacteria
Source: Front Microbiol. 2013 Dec 23;4:407. doi: 10.3389/fmicb.2013.00407 (PMC3870299; doi:10.3389/fmicb.2013.00407)
Supplement: Supplementary file 1 [file Presentation1.PDF]

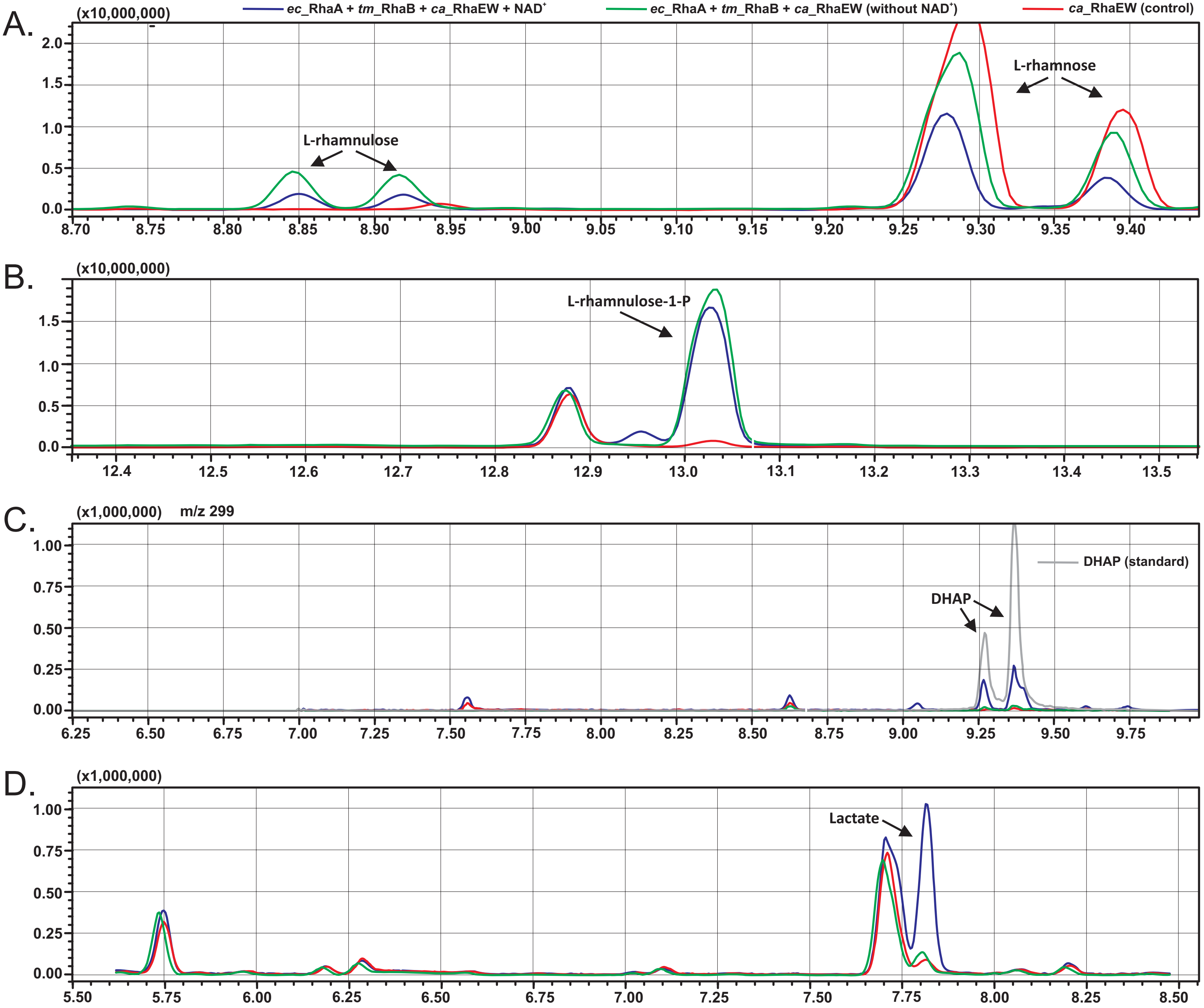

Figure S1. Characterization of reaction products of ec_RhaA, tm_RhaB and ca_RhaEW enzymes by GC_MS. The initial mixture containing L-Rha was incubated overnight with the recominant *c*_RhaA, *tm*_RhaB, *ca*_RhaEW and NAD+ (blue line), resulting in the substantial decrease of two L-Rha-specific peaks and the appearance of new peaks attributed to L-rhamnulose (A), L-rhamnulose-1-P (B), DHAP (C) and lactate (D). Retention time of the DHAP-specific peaks on the chromatogram is identical to retention time of DHAP standard. The same reaction mixture that lacks NAD+, a cofactor of the RhaW dehydrogenase, did not produce the DHAP- and lactate-specific peaks (green line), suggesting that both aldolase and dehydrogenase domains of RhaEW were not active. The control reaction mixture containing L-Rha and ca_RhaEW did not show the appearance of any new peak (red line).
