# Supplementary material for: Comparative genomics and functional analysis of rhamnose catabolic pathways and regulons in bacteria
Source: Front Microbiol. 2013 Dec 23;4:407. doi: 10.3389/fmicb.2013.00407 (PMC3870299; doi:10.3389/fmicb.2013.00407)
Supplement: Supplementary file 2 [file Presentation2.PDF]

A.

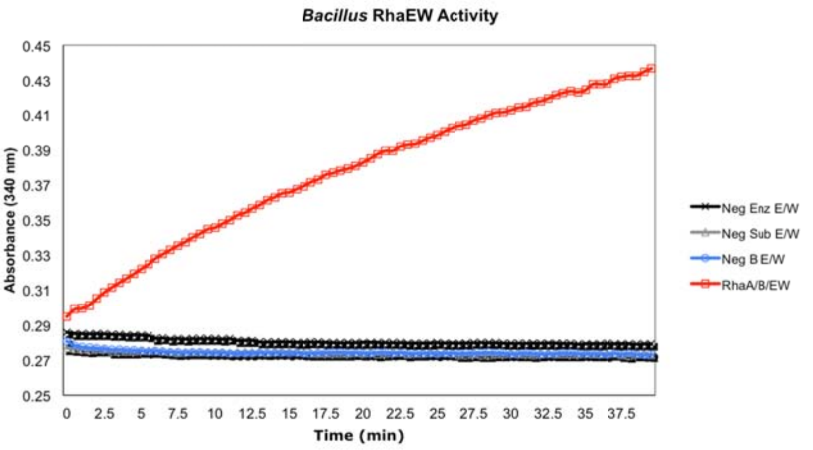

#

B.

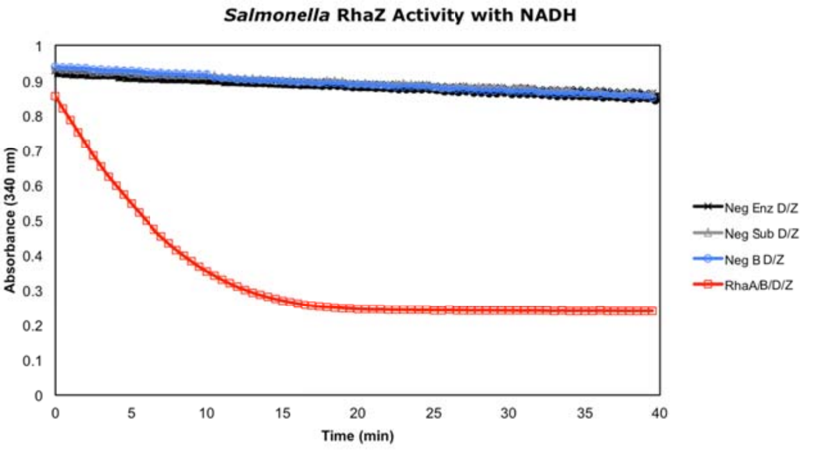

#

C.

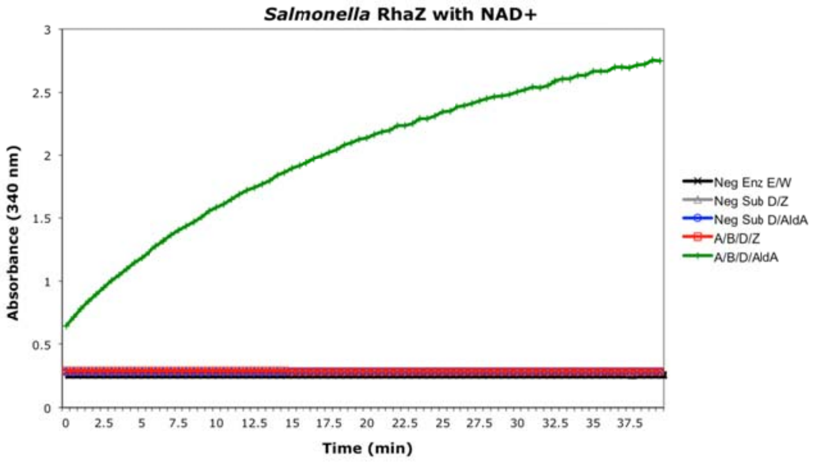

#

Figure S2. Enzymatic activities of *Bacillus subtilis* RhaEW and *Salmonella typhimurium* RhaZ.
(A) *Bs*_RhaEW shows activity on L-Rhamnulose-1-P (RhaE) and L-lactaldehyde (RhaW) in an *in vitro* reconstituted L-Rha utilization pathway. Red squares, RhaEW; Black X, minus RhaEW; Gray Triangles, minus L-Rha; Blue Circles, minus RhaB.
(B) *St*_RhaZ functions as a L-lactaldehyde reductase in L-Rha catabolism, which can be seen as a decrease in absorbance at 340 nm upon conversion of NADH as a cofactor in the production of 1,2-propanediol.
Red squares, RhaZ; Black X, minus RhaZ; Gray Triangles, minus L-Rha; Blue Circles, minus RhaB.
(C) *St*_ RhaZ does not produce L-lactaldehyde in the presence of NAD^+. The *E. coli* enzyme, AldA, produces L-lactaldehyde and serves as a positive control. Red Squares, RhaZ; Green Plus, AldA; Black X, minus RhaZ; Gray Triangles, minus L-Rha with RhaZ; Blue Circles, minus L-Rha with AldA.
