# Supplementary material for: Comparative genomics and functional analysis of rhamnose catabolic pathways and regulons in bacteria
Source: Front Microbiol. 2013 Dec 23;4:407. doi: 10.3389/fmicb.2013.00407 (PMC3870299; doi:10.3389/fmicb.2013.00407)
Supplement: Supplementary file 3 [file Presentation3.PDF]

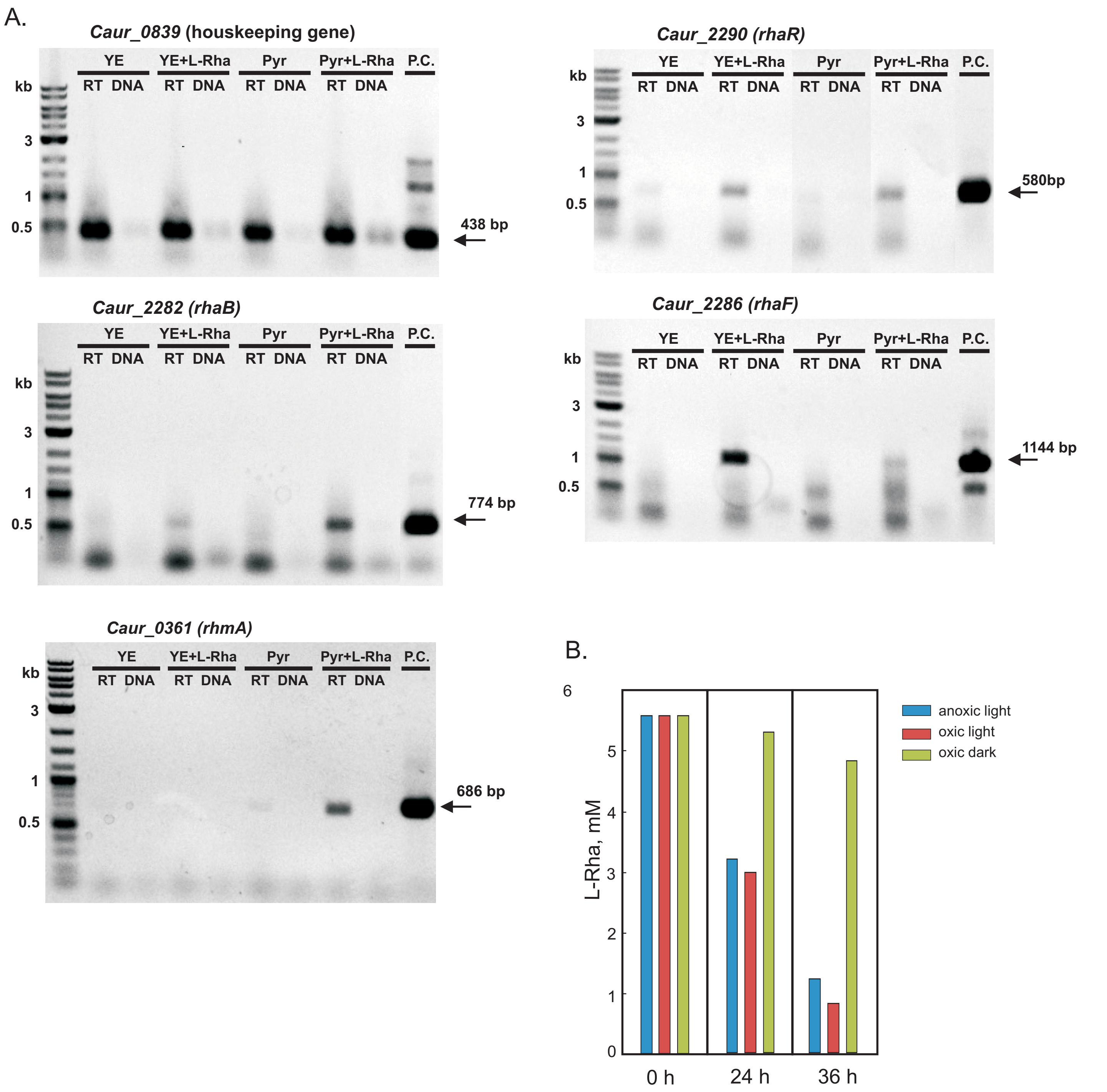

Figure S3. ***In vivo*** **validation of L-rhamnose regulon and utilization in** ***C. aurantiacus*** **J-10-fl.**

A. RT-PCR analysis. Individual transcript levels were measured for three predicted RhaR-regulated genes (*rhaR*, *rhaF*, and *rhaB*), a putative α-L-rhamnosidase gene, *rhmA*, and a housekeeping gene used as a positive control. *C. aurantiacus* was grown in defined medium containing yeast extract (YE) or pyruvate (Pyr) as a carbon source, and supplied with L-rhamnose (L-Rha). RT-PCR was conducted using the Bioline tetro one-step RT-PCR kit. For each reaction one control for DNA contamination was included (started with inactivation of RT-polymerase step). As PCR positive control (P.C.) the whole genome DNA was used. The estimated size of each gene PCR product is indicated.

B. L-Rhamnose consumption by ***C. aurantiacus.*** Culture was grown on L-Rha-supplied defined medium oxic / anoxic and light / dark conditions. Metabolite analysis of L-Rha-containing cultural fluids was performed by HPLC. Values at '0 h' represent the starting concentration of L-Rha (5.6 mM). The '24 h' and ' 36 h' groups represent the concentrations of L-Rha in the culture samples collected 24 and 36 hours after inoculation, respectively.
