# Supplementary material for: Comparative genomics and functional analysis of rhamnose catabolic pathways and regulons in bacteria
Source: Front Microbiol. 2013 Dec 23;4:407. doi: 10.3389/fmicb.2013.00407 (PMC3870299; doi:10.3389/fmicb.2013.00407)
Supplement: Supplementary file 4 [file Presentation4.PDF]

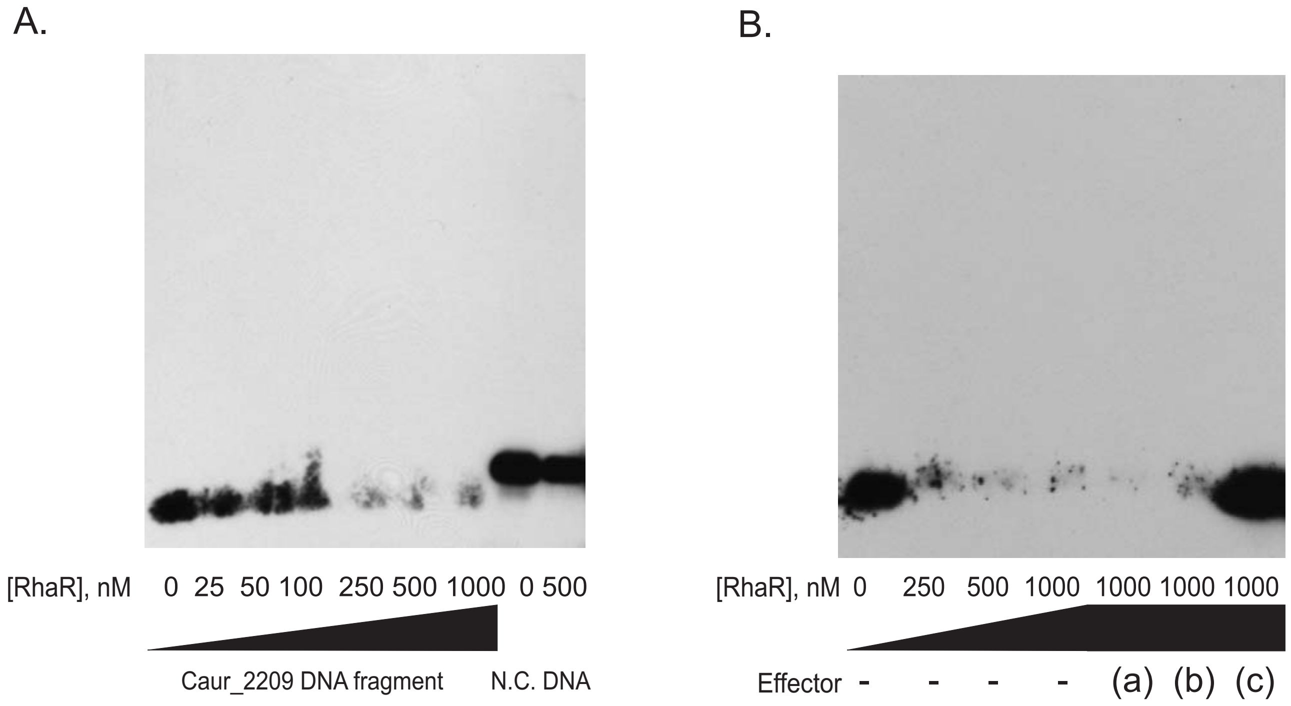

Figure S4. Electrophoretic mobility shift assay (EMSA) to assess the interaction of recombinant RhaR protein from *C. aurantiacus* with its predicted DNA motif and testing of potential effectors of RhaR.

(**A**) Titration of RhaR for binding of upstream DNA fragments of *Caur_2209* (*rhaR*). The biotin-labeled 48-bp DNA fragment (0.2 nM) was incubated at 50ºC with increasing concentrations of the purified RhaR protein. Additional DNA fragment of the *Caur_0003* gene upstream region (200 bp) was used as a negative control. The disappearance of unbound *Caur_2209* DNA band was observed at increasing RhaR protein concentrations.

(**B**) Influence of potential effectors on the RhaR-dependent disappearance of unbound *Caur_2209* DNA band: (a), D-glucose (2 mM); (b), L-rhamnose (2 mM); (c), L-rhamnulose (2 mM). Addition of L-rhamnulose led to re-appearance of the initial DNA band, suggesting the L-Rha catabolic pathway intermediate is an effector of RhaR.
