# Supplementary material for: Comparative genomics and functional analysis of rhamnose catabolic pathways and regulons in bacteria
Source: Front Microbiol. 2013 Dec 23;4:407. doi: 10.3389/fmicb.2013.00407 (PMC3870299; doi:10.3389/fmicb.2013.00407)
Supplement: Supplementary file 5 [file Presentation5.PDF]

A. DeoR family

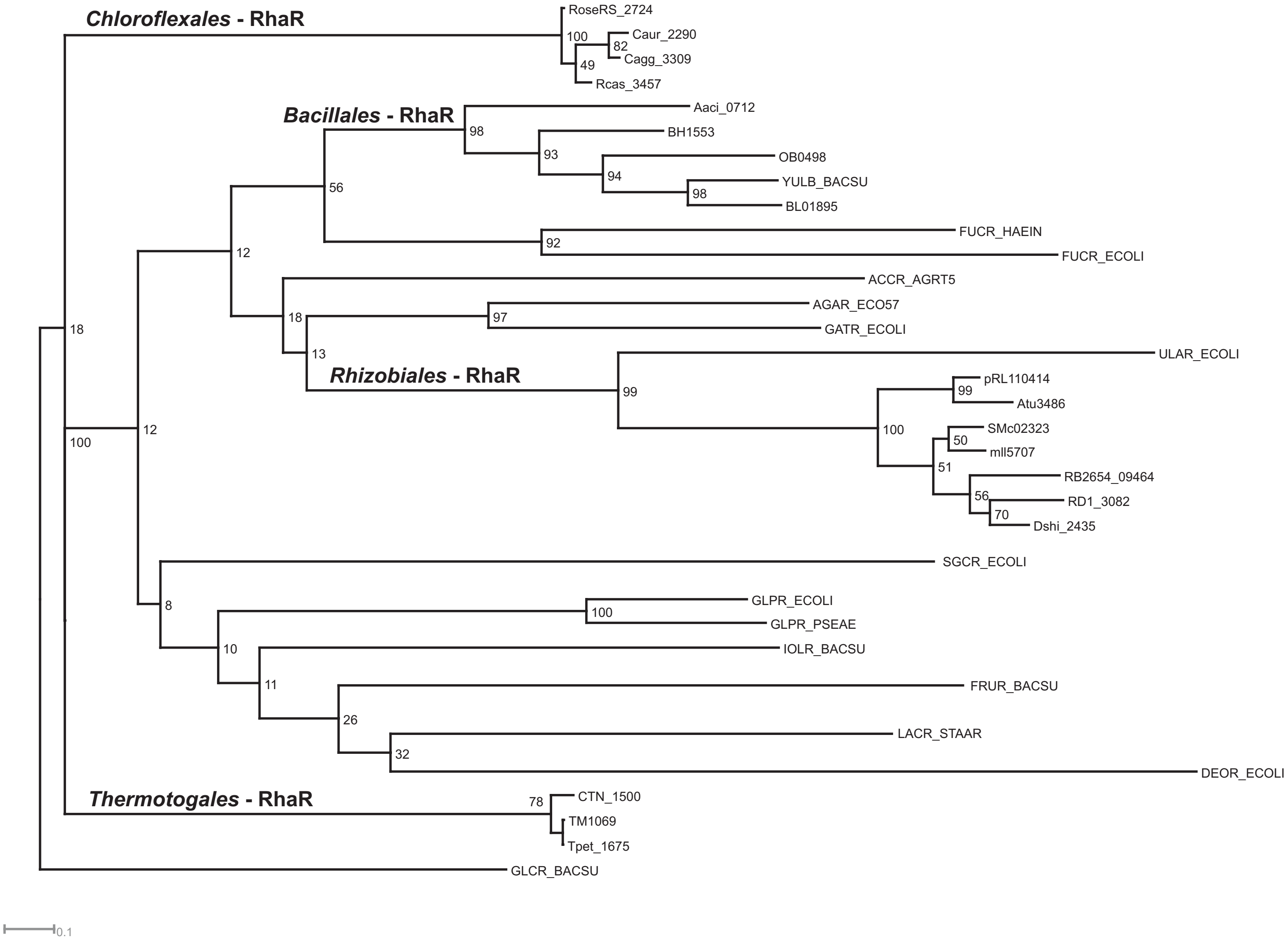

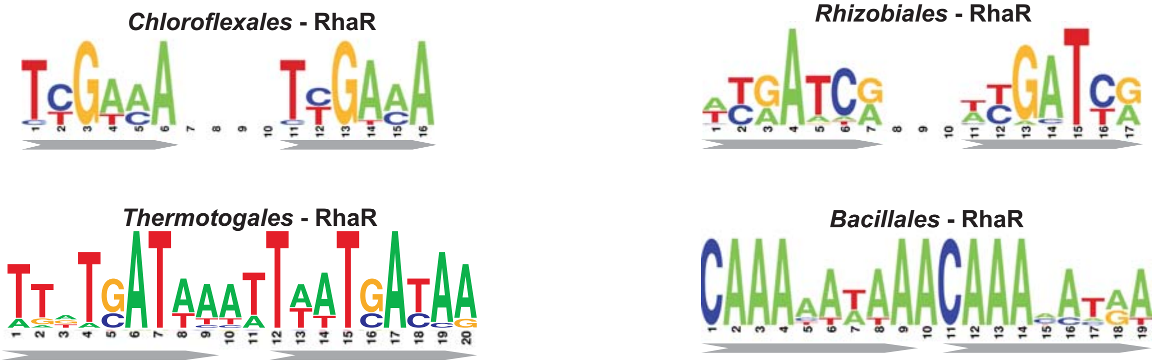

Figure S5. Maximum likelihood phylogenetic trees of transcriptional regulators for L-Rha catabolic pathways. (A) DeoR-family transcriptional factors; **(B)** LacI-family transcriptional factors; **(C)** AraC-family transcriptional factors.

Phylogenetic trees were built using the PhyML server (http://atgc.lirmm.fr/phyml/) (Guindon et al., 2010) using the default parameters and the bootstrap values from 100 replicates. Phylograms were visualized with Dendroscope (Huson et al., 2007). Other previously characterized regulators from the same protein family were collected from UniPROT (http://www.uniprot.org/).

B. LacI family

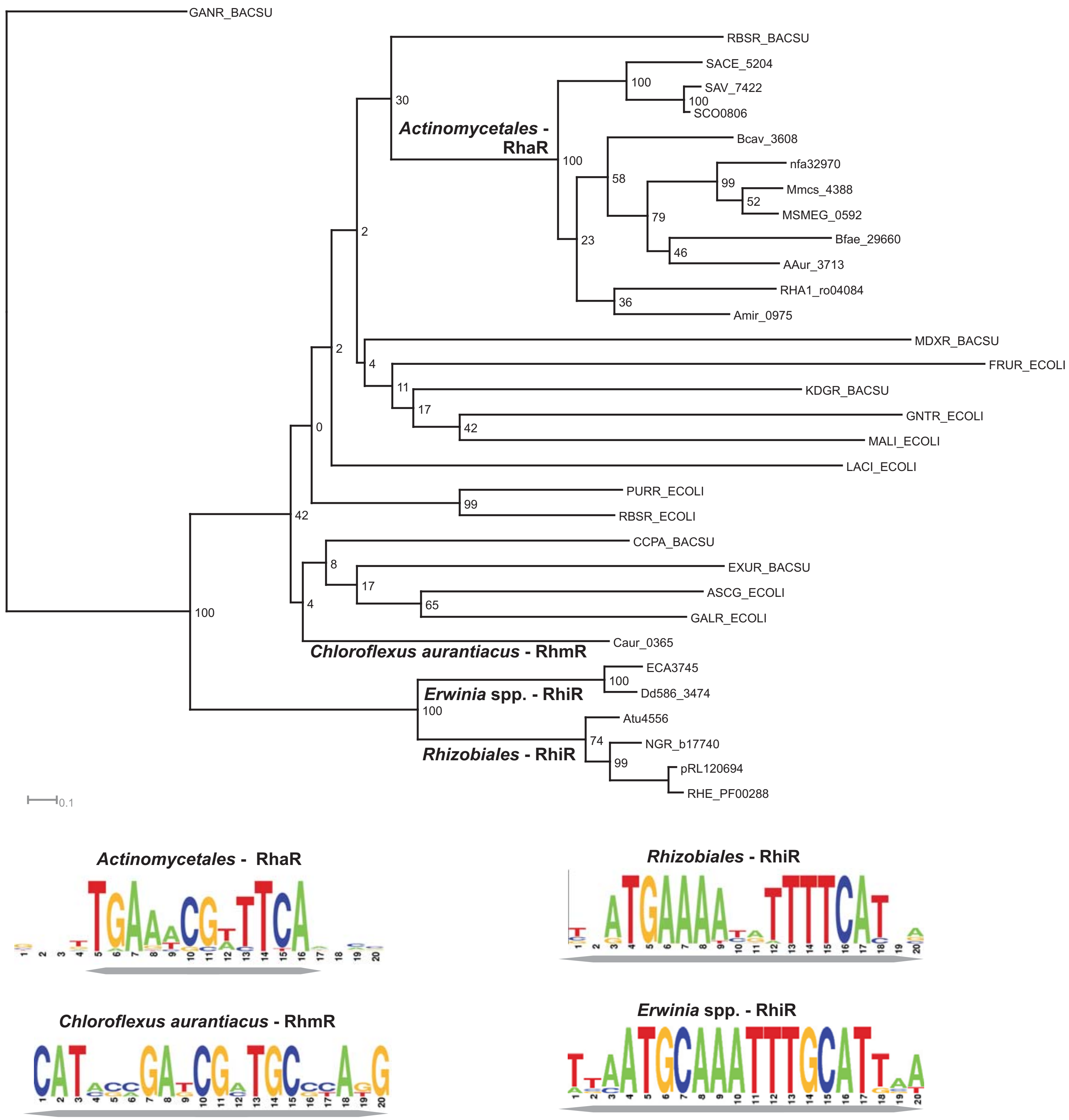

Figure S5. Maximum likelihood phylogenetic trees of transcriptional regulators for L-Rha catabolic pathways. **(A)** DeoR-family transcriptional factors; **(B)** LacI-family transcriptional factors; **(C)** AraC-family transcriptional factors.

Phylogenetic trees were built using the PhyML server (http://atgc.lirmm.fr/phyml/) (Guindon et al., 2010) using the default parameters and the bootstrap values from 100 replicates. Phylograms were visualized with Dendroscope (Huson et al., 2007). Other previously characterized regulators from the same protein family were collected from UniPROT (http://www.uniprot.org/).

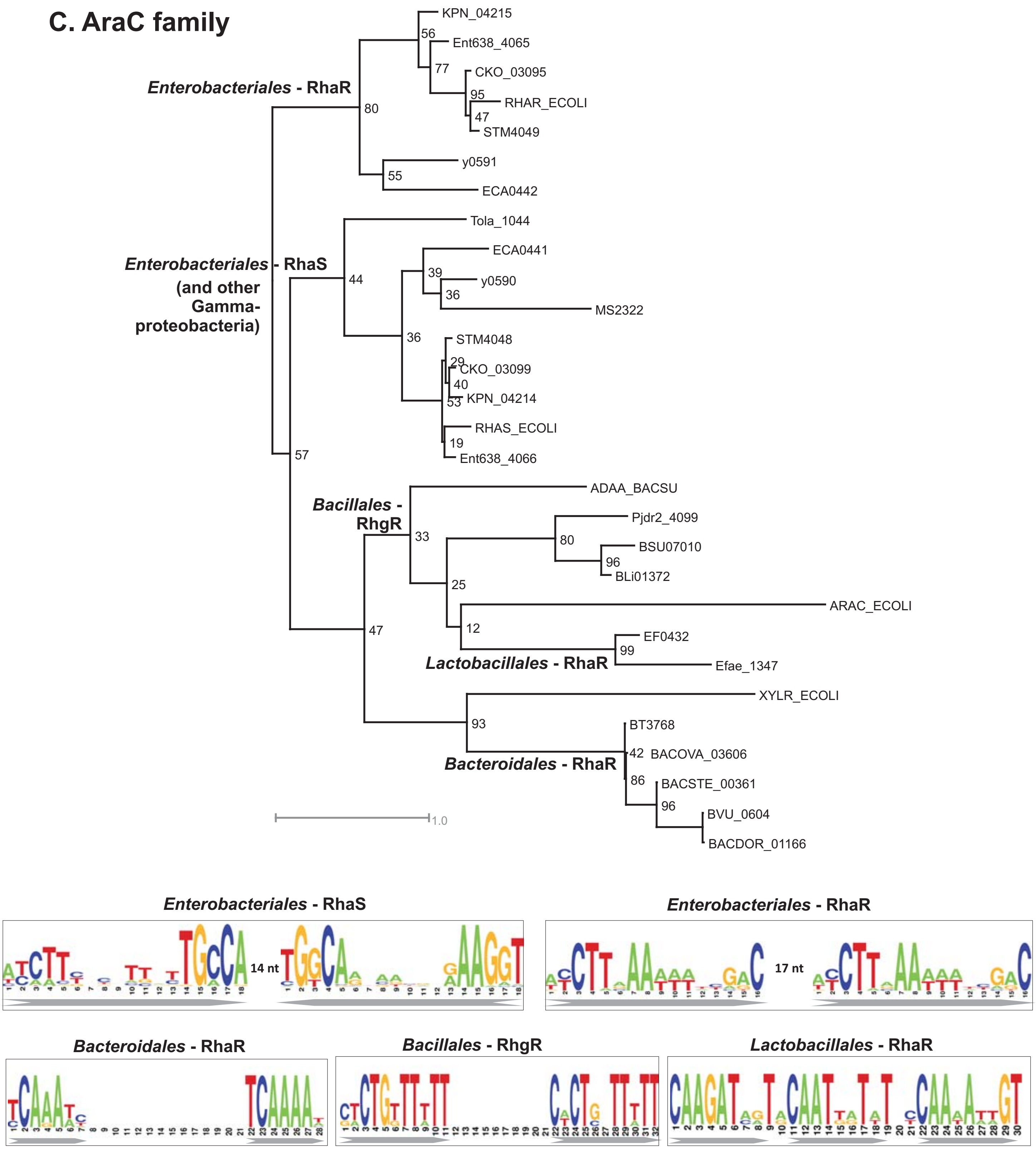

Figure S5. Maximum likelihood phylogenetic trees of transcriptional regulators for L-Rha catabolic pathways. **(A)** DeoR-family transcriptional factors; **(B)** LacI-family transcriptional factors; **(C)** AraC-family transcriptional factors.

Phylogenetic trees were built using the PhyML server (http://atgc.lirmm.fr/phyml/) (Guindon et al., 2010) using the default parameters and the bootstrap values from 100 replicates. Phylograms were visualized with Dendroscope (Huson et al., 2007). Other previously characterized regulators from the same protein family were collected from UniPROT (http://www.uniprot.org/).
