# Supplementary material for: Comparative genomics and functional analysis of rhamnose catabolic pathways and regulons in bacteria
Source: Front Microbiol. 2013 Dec 23;4:407. doi: 10.3389/fmicb.2013.00407 (PMC3870299; doi:10.3389/fmicb.2013.00407)
Supplement: Supplementary file 6 [file Presentation6.PDF]

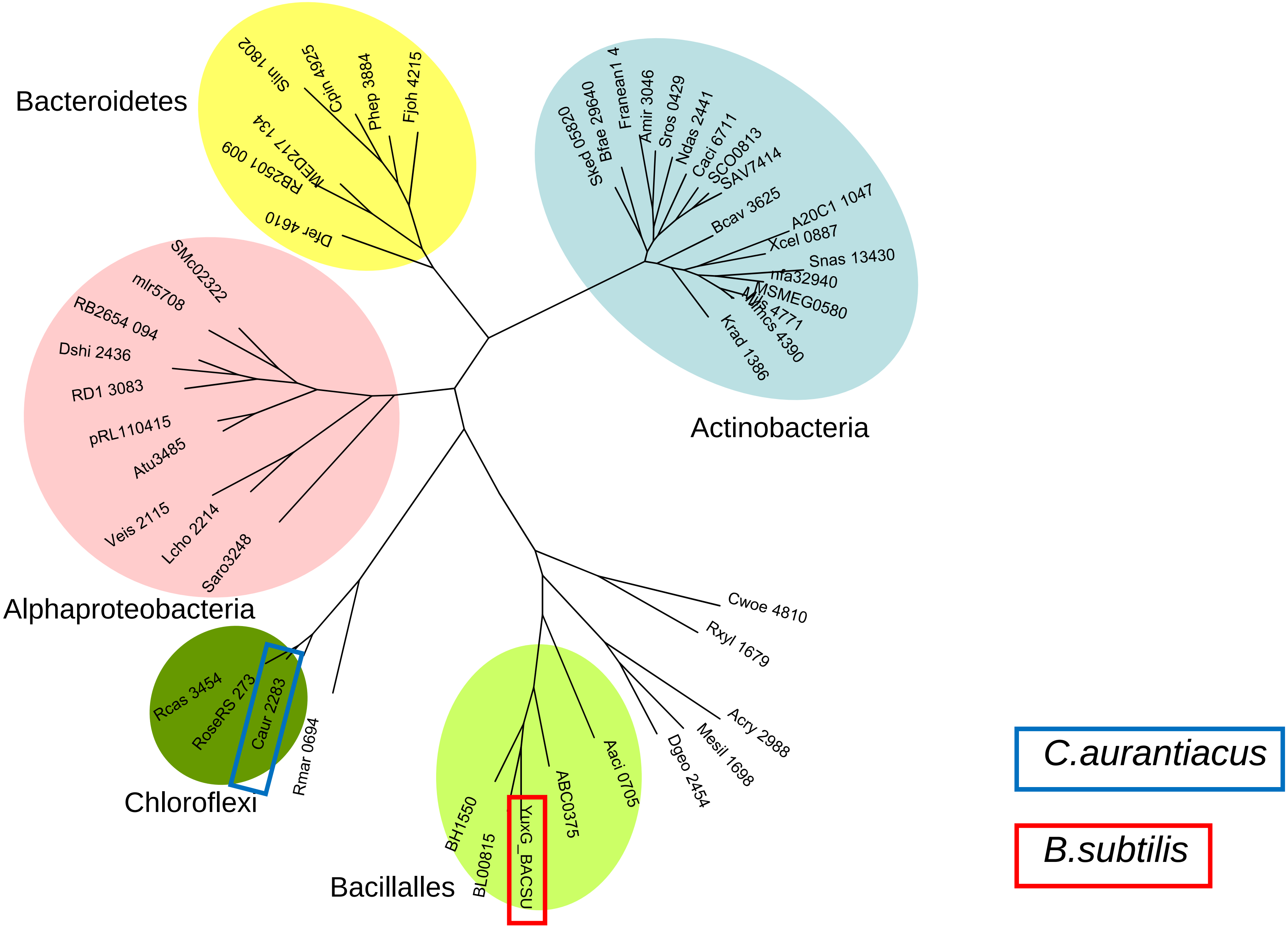

Figure S6. Maximum likelihood phylogenetic tree of the RhaEW proteins. Bifunctional RhaEW proteins contain the L-rhamnulose-1-P aldolase domain RhaE and the L-lactaldehyde dehydrogenase domain RhaW . *rhaEW* genes are always located within the rhamnose utilization gene loci.
