# Supplementary material for: Comparative genomics and functional analysis of rhamnose catabolic pathways and regulons in bacteria
Source: Front Microbiol. 2013 Dec 23;4:407. doi: 10.3389/fmicb.2013.00407 (PMC3870299; doi:10.3389/fmicb.2013.00407)
Supplement: Supplementary file 7 [file Presentation7.PDF]

```
                                                                      **
1gt7_Ecoli_RhaD   1  MQNI-----------------TQSWFVQGMIKATTDAW-LKGWDERNGGNLTLRLDDADIAPYHDNFHQQ   52
1fua_Ecoli_FucA   1  M-------------------ERNKLARQIIDTCLEMT-RLGLNQGTAGNVSVRYQ--------------   35
Caur_2283_RhaE    1  M-NTDNTRFRHVKYGWDAAYAATLDPVARLVYRSNLLGSDQRITNTGGGNTSAKITE-------------   56
Consensus_ss:                              hhhhhhhhhhhhhhhh h         eeeeee

                                            *
1gt7_Ecoli_RhaD  53  PRYIPLSQPMPLLANTPFIVTGSGKFFRNVQLDPAANLGIVKVDSDGAGY--------------------  102
1fua_Ecoli_FucA  36  ---------------DGMLITPTGIPYEKL---TESHIVFID----GNGK--------------------   63
Caur_2283_RhaE   57  ------RDPLTAAPVEVLWVKGSGGDLRTS---TRANFASLEL--RKLRELRTMYLRDPARGPKTAIEDA  115
Consensus_ss:                        eeeee     hhh     hhheeeee

                                             *                       * *
1gt7_Ecoli_RhaD 103  ----------HILWGLTNEAVPTSELPAHFLSHCERIKATNGKDRVIMHCHATNLIALTYVLENDTAVFT  162
1fua_Ecoli_FucA  64  ----------HEE-----GKLPSSEWRFHMAAYQSR-----PDANAVVHNHAVHCTAVSIL--NR---SI  108
Caur_2283_RhaE  116  MVDLYPHCTFNLN-----PRASSIDTPLHAFI----------PYRHVDHMHPNAVIAIAASRNG------  164
Consensus_ss:                               hhhhhhhhhhhh          eeeee  hhhhhhhh

                                                                          *
1gt7_Ecoli_RhaD 163  RQLWEGSTECLVVFP-DGVGILPWMVPGTDEIGQATAQEMQ---KHSLVLWPFHGVFGSGPTLDETFGLI  228
1fua_Ecoli_FucA 109  P---AIHYMIA-AAGGNSIPCAPYATFGTRELSEHVALALK---NRKATLLQHHGLIACEVNLEKALWLA  171
Caur_2283_RhaE  165  ------ERLTREIY-GDEVIWMPWQRPG-FDLGLKLEQICREHPQAKGVILGGHGLINWADDDQECYERT  226
Consensus_ss:              hhhhhhhh    eeee       hhhhhhhhhh       eeeee   eeeee   hhhhhhhh

1gt7_Ecoli_RhaD 229  DTAEKSAQVLVKVYSMGGMK-------QTISREELIALGKRF----------------GVTPLASALAL-  274
1fua_Ecoli_FucA 172  HEVEVLAQLYLTTLAIT-DPV------PVLSDEEIAVVLEKF----------------------------  206
Caur_2283_RhaE  227  LDLIERAARYIEAHDRGEAT-FGGARYDALPFEQRRSVLAAILPWLRGQISQQRRFIATIQDDATTLRFV  295
Consensus_ss:        hhhhhhhhhhhhhhh                hhhhhhhhhhh                     hhhh
```

Figure S7. Multiple alignment of *C. aurantiacus* RhaE domain and the structurally characterized class II aldolases RhaD and FucA from *E. coli*. Residues involved in catalysis are marked by asterisk and highlighted. Residues involved in zinc binding are in green. Residues interacting with the phosphate group of the sugar are in magenta. The catalytically active Glu residue, which is substituted by Asp in RhaE, is in yellow. Consensus secondary structure elements are indicated below: h, alpha-helix; e, beta-strand.
