# Supplementary material for: Comparative genomics and functional analysis of rhamnose catabolic pathways and regulons in bacteria
Source: Front Microbiol. 2013 Dec 23;4:407. doi: 10.3389/fmicb.2013.00407 (PMC3870299; doi:10.3389/fmicb.2013.00407)
Supplement: Supplementary file 9 [file DataSheet2.DOC]

**Supplemental Table S2. Primers and restriction sites used for gene cloning and RT-PCR.**

1. **Gene cloning.**

| Gene | Organism | Plasmid | Sites | Primers^1^ |
| --- | --- | --- | --- | --- |
| *Caur_2283 (rhaEW)* | *Chloroflexus aurantiacus* J-10-fl | pODC29  N-His6 tag | NcoI/  SalI | F-5’- gggccATGGACACAGATAACACTCGGTTTCG;  R-5’- gggtcgacTCAACGCAGGAAGGCTTCGTGG |
| *Caur_2290 (rhaR)* | *Chloroflexus aurantiacus* J-10-fl | pSMT3  N-His6-SUMO tag | BamHI/  HindIII | F-5’- gaattatggatccATGACAGATCACAGCAATGATG;  R-5’- catattaagcttCTACACAATCGTGATCGGAAAAAAACC |
| *STM4044 (rhaZ)* | ***Salmonella enterica* serovar Typhimurium LT2** | pPROEX-HTb  N-His6 tag | BspHI/  XhoI | F-5’-gtcatctcATGAGCTTTATGTTGGCACTGCCA;  R-5’-gactacctcgagTCATAACGCCTCCAGGTACA |
| *STM4045 (rhaD)* | ***Salmonella enterica* serovar Typhimurium LT2** | pPROEX-HTb  N-His6 tag | NcoI/  XhoI | F-5’-gtcatcccATGgAAAATATTACCGATTCCTGGTTC;  R-5’- gactacctcgagTCAGTACAGCGCTACGGCG |
| *yuxG (rhaEW)* | *Bacillus subtilis* | pPROEX-HTb  N-His6 tag | NcoI/  XhoI | F-5’-gtcatcccATGGTGAAACATATATGGGATTCAG;  R-5 gactacctcgagcTAACGCGTAAAAGCAGC |

^1^Introduced restriction sites are underlined; nucleotides not present in the original gene sequences are shown in lowercase.

**B. RT-PCR.**

| Gene | Organism | Primers |
| --- | --- | --- |
| *Caur_2290 (rhaR)* | *Chloroflexus aurantiacus* J-10-fl | F-5’- CGGTGTTTCTGAGGTGACGA;  R-5’- CGGACAAAAGCTGGTCAACG |
| *Caur_2286 (rhaF)* | *Chloroflexus aurantiacus* J-10-fl | F-5’- GTTGTTCTGCTGATCGTCGC;  R-5’- ACGTGATCGGAGCATTACCC |
| *Caur_2282 (rhaB)* | *Chloroflexus aurantiacus* J-10-fl | F-5’- ACGAGCAATCAAGCCGGTTA;  R-5’- TCGCATCCCAACTCAGGTTC |
| *Caur_0361 (rhmA)* | *Chloroflexus aurantiacus* J-10-fl | F-5’- GTGCGTCTTCTTGAGTGGGA;  R-5’- GCAGAGGGGCCTAACACATT |
| *Caur_0839 (control)* | *Chloroflexus aurantiacus* J-10-fl | F-5’- TGATCTTAGAGGCAACCGCC;  R-5’- CATACGCATCGGTACGGTCA |

**C. DNA fragments for EMSA.**

| Gene | Organism | Length | Sequence |
| --- | --- | --- | --- |
| *Caur_2290 (rhaR)* | *Chloroflexus aurantiacus* J-10-fl | 48 bp | 5′-gggggTTCGTAAAATCTCGTAAGTACTTGACAAATCTTGAAAAggggg-3′ |
| *Caur_0003* | *Chloroflexus aurantiacus* J-10-fl | 200 bp | 5′-Ggcaggcatccgtagagcgctctacgataacgatttggcaaaactttaccgtatctttaatctctgatcacatttattcgctattgtgaaaatatccacagtgcaatggtttgacacagatacgcggcgcagcctataatcgaagcatccgcaatgtaagcaccccgccgcatggattcgtaacaaggaggttcgacaca-3′ |
